# Detection of communities with Naming Game-based methods

**DOI:** 10.1371/journal.pone.0182737

**Published:** 2017-08-10

**Authors:** Thais Gobet Uzun, Carlos Henrique Costa Ribeiro

**Affiliations:** Dept. of Computer Science, Aeronautics Institute of Technology, Sao Jose dos Campos, Sao Paulo - Brazil; Universite Toulouse 1 Capitole, FRANCE

## Abstract

Complex networks are often organized in groups or communities of agents that share the same features and/or functions, and this structural organization is built naturally with the formation of the system. In social networks, we argue that the dynamic of linguistic interactions of agreement among people can be a crucial factor in generating this community structure, given that sharing opinions with another person bounds them together, and disagreeing constantly would probably weaken the relationship. We present here a computational model of opinion exchange that uncovers the community structure of a network. Our aim is not to present a new community detection method proper, but to show how a model of social communication dynamics can reveal the (simple and overlapping) community structure in an emergent way. Our model is based on a standard Naming Game, but takes into consideration three social features: trust, uncertainty and opinion preference, that are built over time as agents communicate among themselves. We show that the separate addition of each social feature in the Naming Game results in gradual improvements with respect to community detection. In addition, the resulting uncertainty and trust values classify nodes and edges according to role and position in the network. Also, our model has shown a degree of accuracy both for non-overlapping and overlapping communities that are comparable with most algorithms specifically designed for topological community detection.

## Introduction

One property that many real networks seem to share is community structure, when nodes organize themselves in sub-units—or communities [1]. These communities, depending on the nature of the problem, can be considered as almost independent compartments of a graph [2], playing different roles and influencing the functionality of the system. This way, the identification of these sub-units, the building blocks of the network, can provide crucial information about the system in question [2], given that the existence of community structure itself indicates that the nodes of the network are not homogeneous, but divided into classes [3]. Many algorithms of Community Detection have been presented in the literature in the last years, some of them having excellent accuracy and performance. However, most of those methods focus only on the topological features of the network and use heuristics and global information to obtain the best set of communities, even though communities are formed in a natural emergent way in real networks. This phenomenon can be seen by the existence of groups in social networks that share the same city, same routine, same language, same opinions, etc. Those groups, however, tend to dissolve or get stronger naturally, due to changes in the shared aspects. One of these aspects, that will be studied in this work, is the linguistic interaction or opinion exchange among agents in a network.

Language has been investigated by sociolinguistics to influence community formation [4][5]. In [5], Gumperz defines a *speech community* as a group of humans that have interactions frequently, using a shared body of verbal signs that emerges through living and interacting together. These verbal interactions result from a social process in which utterances are selected according to socially recognized norms and expectations.

In this work, we will interpret a community as a group of individuals who share a linguistic convention that was developed in an emergent way, through communications dependant on some social norms, in parallel with Gumperz definition. To do so, we use a model of language interactions—the Naming Game (NG) [6]—that was first developed to show how linguistic conventions are formed in an emergent way. The model was originaly applied to robotic agents that were then able to develop a language to describe themselves and the environment. The Naming Game, however, treats every individual and relationship as equal in every step of the game, regardless of any social norm. In this work, we will insert three social features on individuals playing the Naming Game to guide their interactions and reveal the speech communities present on the system. In this way, we hypothesize that the topological communities of the network will be instantiations of the speech communities that emerge as a result of a language dynamics in that network.

The first social feature inserted in the model is *trust*: we believe that what links people together is not only their topological proximity (or geographical proximity in a social sense) but their shared linguistic conventions: if they can communicate and agree with each other they will trust each other more than if they cannot communicate, and these levels of trust will guide the next communications. Trust will be used, then, as a social feature that influences the language interactions. It will be modeled as the weight of the edge that links both individuals (similarly to the definition of trust in [7]), in a variation of the NG called *Naming Game with Adaptive Weights* (NG-AW) [8].

The second inserted social feature is *uncertainty* [9]: the value of trust is based on communications that already happened, and will guide the preference for the next interactions. Indeed, in a social scope opinions about others are based on the trust already developed. However, these opinions can change in time and the (at first) considered trustworthy relations can be deceitful. For this matter, we introduce a factor for modeling the uncertainty(*ϵ*_*i*_) an individual would have in its constructed trust which naturally decays as the individual gathers knowledge on who to trust based on the outcomes of earlier communications. In our model, the probability of communication through an edge *p*(*i*, *j*) would then be proportional to *trust*_*ij*_ + *ϵ*_*i*_.

Given that both trust and uncertainty are based on the outcome of a communication, which, in turn, depends on the actual utterance made in the interaction, the last inserted social feature is the *utterance preference* [10], that rules the choice of a subject for the communication. We argue that an individual would prefer to communicate with an utterance that has been understood before. In our model, this corresponds to the agent choosing to communicate preferentially using a word that it believes will lead to a successful interaction. We call this feature *opinion preference*, since the Naming Game can be interpreted as an opinion dissemination model and this is how we will generally refer to it in the rest of the paper.

In order to investigate the similarity between the speech communities and the existing topological communities, we use the Naming Game-based model we developed as a Community Detection algorithm, where utterances are interpreted as propagating labels and groups sharing the same language conventions are interpreted as communities. There are many similarities between the proposed model and a label propagation algorithm: both are based on local interactions; start with all nodes in the same state; and the resulting labels (or words) tag the existing communities in the network, each being represented by a unique label (word). In fact, label propagation algorithms were part of the motivation for this work.

Our first contribution with this paper is showing that, with the insertion of each social parameter, the speech communities will correspond more and more to the topological communities of the analyzed networks. More specifically, we show how a language agreement dynamics on a social network can reveal its modular structure by reaching a meta-stable state of groups of nodes sharing the same word, representing the existing communities. We measure how well the speech communities match the real communities by comparing them as we would do for a community detection algorithm. The meta-stable state happens in non-convergent executions, when words get trapped inside the communities. Our second contribution is to show that the model with three social features is robust for different networks, when we analyze the influence of the network parameters in the dynamics. Our third main contribution is applying the model to networks with overlapping communities and realizing that the model is also fit for such cases.

The evolution in time of each incorporated social feature studied here—namely trust, uncertainty and utterance preference—results in the self-organization of edges, nodes and words, respectively. In this way, the presented Naming Game model not only uncovers the existing communities in the network, but also organizes network components in a natural emergent way.

This paper is organized as follows: the next section—Materials and Methods—presents the metrics to analyze the detection of communities, and the benchmarks for the construction of the artificially generated networks used for the experimental evaluation. The section also details the minimal Naming Game and three variations, each incorporating a social feature. At the end of the section we introduce the last variation for networks with overlapping communities. In the Results section we first apply the three methods as Community Detection algorithms and analyze their behaviours, showing that the incorporation of the features leads to better community detection. Later in the section, we vary the construction parameters of the network topology and analyze the impact on the community detection accuracy. We also test the algorithms in real-world networks. At the end of this section, we analyze the accuracy of the model with the three features for overlapping community detection, along with an analysis on the influence of network construction parameters. Finally, the Discussion and Conclusions section presents the main conclusions of this research, suggestions for future work and some additional discussion about the implications of the results that can also lead to future work.

## Materials and methods

### Community detection metrics and benchmarking

Formal definitions of community have been extensively discussed and none is universally accepted, as it often depends on the application [2]. In this work, the classical definition of community as a group of nodes that have more edges between them than edges linking those nodes to the rest of the network will be used. We define *external edges* as edges between two nodes from different communities and *internal edges* as edges between nodes in the same community. Thus, the aim here is to find a set of groups that have more internal edges than external ones.

The set of communities defines a *partition* where each node is assigned to one and only one community. However, in real networks agents can belong to different communities such as family, work, church, etc. The division of a graph into overlapping communities is called a *cover*. Finding partitions and covers are two research branches in Community Detection.

Thus, the aim of any community detection algorithm is to find a partition or cover that represents the communities in the network. In order to evaluate the quality of a given algorithm, we must therefore analyze the community structure found by it, relatively to some ideal setting. In this work, we use for this purpose artificial networks, whose optimal community structure is known in advance. We then compare the found partition (or cover) with this one using the *Normalized Mutual Information* (NMI), a measure of similarity of partitions based on Information Theory [11, 12]. The NMI between two partitions (or covers [13]) lays between 0 and 1, corresponding to independent or identical partitions, respectively.

In real systems, however, one needs a measure to qualify a found partition regarding the correct detection of existing communities that are not known a priori. In order to evaluate a found partition, Newman and Girvan [14] defined a partition quality function called *modularity*, based on the idea that the existence of communities or modules is revealed by a comparison between the density of edges of a random graph and the density of edges of the actual graph [2]. The modularity value will be used in the analysis of real networks.

The artificially generated networks used in this work are based on the LFR benchmark [15], a recently developed and already one of the most used community detection benchmarks. It is based on the fact that real-world networks are characterized by heterogeneous distributions of node degree, with tails that often decay according to a power law. Likewise, it also takes into account the fact that different communities don’t always have the same size: in fact, the distribution of community sizes of real networks can also be approximated by a power law [15]. Based on that, the benchmark generates networks with power law distributions of both degree and community size, with exponents *τ*_1_ and *τ*_2_, respectively. The parameter responsible for the community structure in the network is called *mixing parameter* *μ*, and indicates the fraction of external links each node will have. Consequently, each node will have (1−*μ*) of its edges with nodes from its own community.

The LFR benchmark was extended to generate networks with overlapping communities—where each node is allowed to have multiple memberships—whilst maintaining those features [16]. The *μ* parameter is replaced by the *topological mixing parameter*, that represents the portion of neighbors of a given node that share no memberships with it. The network generator for for different networks built according to the LFR benchmark can be downloaded freely from https://sites.google.com/site/santofortunato/inthepress2.

### The Naming Game

The original Naming Game model was developed by Luc Steels [17] as a way to test the hypothesis that language is an autonomous adaptive system that forms itself in a self-organizing way, as opposed to beliefs of the scientific community that language was a static system of rules. In 2006, Baronchelli *et. al*. [6] proposed the minimal version of the Naming Game to reproduce the fundamental features and main results of the original Naming Game using simpler rules.

The minimal Naming Game is a model that can capture microscopic features of an agreement process occurring in a network, establishing a lower bound in complexity and performance. The game is played by *N* identical agents lying on a given network topology. Each agent has a local memory or inventory that starts empty. The game ends when a consensus or convergence state where all agents have only one and the same word in their memories, is reached.

The Naming Game rules are described in Algorithm 1. These simple rules trigger three mechanisms: an uploading mechanism responsible for introducing new words into the system; an overlapping mechanism that allows the dissemination of various words between agents, and an agreement mechanism that allows the deletion of unnecessary words. With these mechanisms the system has a disorder-order transition towards convergence [18].

**Algorithm 1:** Naming Game

**for** *each time step* **do**

 One agent is randomly chosen as a speaker;

 Among its neighbors, one agent is chosen to be the listener;

 **if** *the speaker’s memory is empty* **then**

  the speaker invents a word;

 **else**

  the speaker selects one of the words in his inventory;

 **end**

 The speaker transmits the word to the listener;

 **if** *the listener has the word in his memory* **then**

  the communication is a *success* and both speaker and listener delete all the words from their memories except the transmitted word;

 **else**

  the communication is a *failure* and the listener adds the transmitted word to his inventory

 **end**

**end**

**Fig 1:**
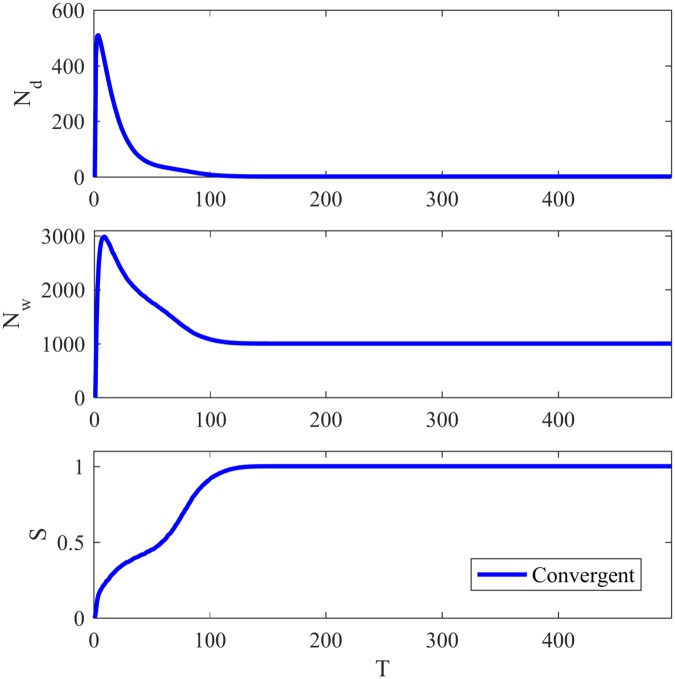
Basic features of the Naming Game. Evolution of the number of different words *N*_*d*_, total number of words *N*_*w*_ and success rate *S* for the minimal NG in a LFR network with 1000 agents and *μ* = 0.3.

In order to analyze the Naming Game, some classical measures are usually monitored over time: the total number of words in the network *N*_*w*_, the number of different words in the network *N*_*d*_, and the successful communications rate *S*. *N*_*w*_ informs the memory required in the game, *N*_*d*_ represents the lexical coherence of the developed language and *S* represents the probability of observing a successful communication at a point in time. Fig 1 shows the evolution of *N*_*d*_, *N*_*w*_ and *S* over time for a LFR network with 1000 agents, *μ* = 0.3, 〈*k*〉 = 20, *τ*_1_ = 2, *τ*_2_ = 1, *k*_*max*_ = 50, size of communities *C*_*min*_ = 10, *C*_*max*_ = 50. We adopted the unit *T* = *N* communication attempts as defined in [8]. All simulations in this work were executed and averaged over 100 games.

At the first steps of the game, many words are invented in different nodes of the network until there are N/2 different words, so one in each two agents invents a word. After some interactions, many words are eliminated due to an increase in the success rate, and a large number of small clusters sharing the same word develop. As time passes by, more words are eliminated and smaller clusters disappear, giving rise to the emergence of larger clusters and eventually leading to one large cluster comprising all agents. In other words, through pairwise communications agents slowly reconcile their “differences” and at the end all share the same word, *i.e.*, converge.

Although the Naming Game was originally built to describe the formation of language, it has been used as a model for language spreading, opinion dynamics and voting dynamics/prediction [19, 20]. In this work, we will interpret the Naming Game as a model for opinion dynamics.

The convergence of minimal Naming Game is guaranteed for various network topologies, varying in the time to reach convergence and in the memory required to store all words. In an interesting way, [21] applies it to a real high-school friendship network with extremely high community structure. For such network, the consensus state is not always reached, but sometimes the system gets trapped on a meta-stable state with coexisting clusters of nodes sharing the same word. These clusters revealed the communities in the friendship network, separating students among different ethnic lines and from high-school and middle-school. The state of non-convergence was not used to detect communities in [21], but *committed agents* were inserted to reach convergence, as a variation of the Naming Game.

Due to the non-determinism of the game, the meta-stable state that revealed the existing communities only happens in a few executions, mostly reaching the convergence state. If external edges are randomly chosen many times at the first steps of the game to host a communication, the probability of non-convergence decays drastically.

This study shows that, in a high community structure network, the Naming Game can reveal the underlying existing communities whenever there is no convergence. However, such behavior happens only with some probability, depending on the community structure of the network. In fact, in a network with high community structure (LFR *μ* = 0.2 and N = 1000) the non-convergence state was reached in less than 33% of the executions. In networks with *μ* ≥ 0.3, convergence is always reached. To detect communities in real world networks, a NG-based approach must therefore allow, with high probability, for the emergence of coexisting clusters sharing the same words, correctly representing different communities.

### Community detection methods based on the Naming Game

Having in mind the conjecture that a Naming Game-based approach can disclose communities in a network, in this subsection we explore three algorithms that aim at naturally detecting communities on the underlying topology through communication interactions, each incorporating a particular social feature.

#### The Naming Game with adaptive weights

The Naming Game with Adaptive Weights (NG-AW) [8] is a NG model of an adaptive weighted network. The weight of an edge between two nodes is the proportion of successful interactions they had so far, thus changing over time. In this way, a pair of nodes with high rate of successes would be linked by a stronger connection, and so on. In this work, this weight will be interpreted as a measure of *trust* between agents. All weights start and remain equal to 0 as long as the edge’s number of attempts is 0.

NG-AW offers the possibility of non convergence even in fully connected networks—a state we achieved in a minimal NG only if the underlying network had high community structure—depending on an input parameter *ϵ*. The information flow changes the edge weights as the game is played, weakening the edges with low success rate and reinforcing those with high success rate, changing the network structure based on opinion spreading. NG-AW operates according to Algorithm 2.

The only difference between this variation and the minimal Naming Game is in the choice of the listener. This choice depends on the edge’s communication history (trust) and on a parameter *ϵ*, whose value is given *a priori* and that can be set to result in either convergence or non-convergence of the game. In fact, this is one of the results presented in [8]: the empirically observed emergence of two different behaviors (convergence and non-convergence) depending on *ϵ*.

**Algorithm 2:** Naming Game with Adaptive Weights

**Input**: *ϵ* value;

**for** *each time step*: **do**

 One agent *i* is randomly chosen as a speaker;

 The agent *i* chooses his listener *j* among his neighbors with probability

$$\begin{array}{c} p_{ij}=\frac{w_{ij}+\epsilon }{\sum_{k=neighbors}\left(w_{ik}+\epsilon \right)} \end{array}$$ (1)

where $w_{ij}=\frac{Successes_{ij}}{Attempts_{ij}}$

 **if** *the speaker’s memory is empty* **then**

  the speaker invents a new word;

 **else**

  the speaker selects randomly one from his inventory;

 **end**

 The speaker transmits the word to the listener;

 **if** *the listener has the word in his memory* **then**

  the communication is a success and both speaker and listener delete all the words from their memories except the transmitted word;

 **else**

  the communication is a failure and the listener adds the transmitted word to his inventory

 **end**

**end**

**Fig 2:**
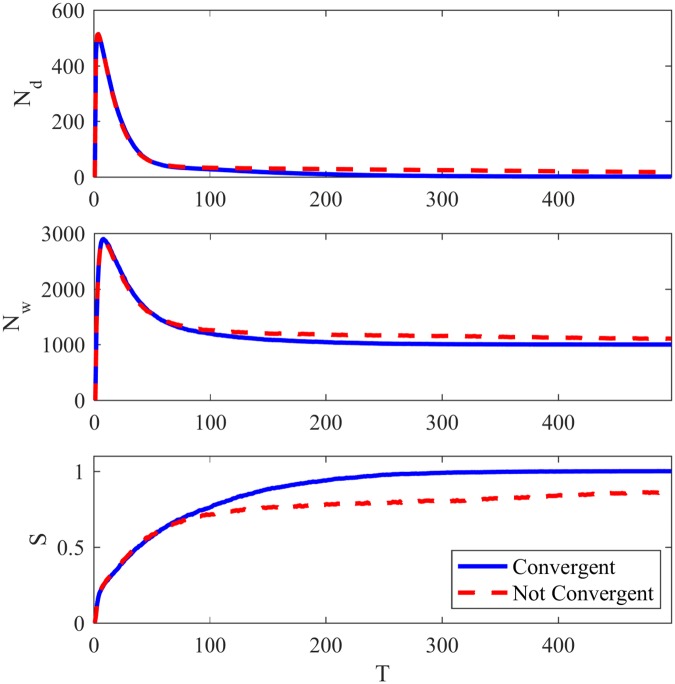
Basic features NG-AW. Basic Features for the Naming Game with adaptive weights.

With a relatively large value of *ϵ* ($\epsilon =\sqrt{\frac{10^{-5}}{N}}$), the system behaves similarly to the minimal Naming Game, achieving consensus in all tests in a fully connected network. With a small *ϵ* ($\epsilon =\sqrt{\frac{10^{-9}}{N}}$), the system enters a multi-word regime, with success rates near to one and several different words coexisting in the network. However, when applied to networks with high community structure, both behaviors can be observed in different executions of the game with the same *ϵ*, as shown in Fig 2, where the game is played in the same LFR network with N = 1000 and *μ* = 0.3, and *ϵ* is set as 10^−0.5^.

In this way, NG-AW will have a convergence probability that indicates the rate of executions that reached consensus, and this probability will be dependant on *ϵ*. It is interesting to point out that, as the total number of words converges to *N*, the number of different words converges to (a number close to) the number of communities, corresponding to the communities assigned to the agents.

As the weight represents the edge’s success rate, when NG-AW is applied to networks with community structure, external edges will have small weights during the game, since they receive different words from two or more different clusters. If communication flows through large weight edges, interactions will take place mostly inside communities, restricting communications between communities. This will result in a multi-word regime with higher probability, and the possibility of this state of non convergence is a key aspect for community detection. As will be shown in the next section, the incorporation of trust not only corresponds to a more realistic situation but also improves the revelation of the communities present on the network.

#### The Naming Game with Local Exploration Factor—NG-LEF

As we saw in last subsection, in NG-AW, the adaptive weights turn communications more frequent inside communities, when compared to the minimal NG. The *ϵ*-adding strategy in NG-AW is mostly used so that, in the word spreading phase of NG, agents do not restrict their communications to neighbors with whom they had successes in the first phase. If they do, redundancy will never be enough to delete all unnecessary words: with *ϵ* close to 0 only a few edges host communications conducted randomly at the beginning of the game. The dissemination happens only through those edges, never allowing the system to gather enough community information about the network. Such information lies in the redundancy of words inside the memories.

In a social scope, we can interpret *ϵ* as a measure of uncertainty of the agent in her/his knowledge about the environment. Thus, an agent chooses the listener based on a deterministic part—that is, his built trust *w*—biased by a random factor, the global fixed *ϵ* value, that will be interpreted as the uncertainty of the agent in that trust. Interpreting it as such, in NG-AW all agents would have the same uncertainty in communication, even after building different trust values with different neighbors in late stages of the game. However, we can speculate that communicating agents can behave differently in this aspect. Considering the natural social domain, a person would need time to know the environment and be sure who to talk to and who to trust. An average person would not trust another one only by agreeing with him/her a few times, but would be less uncertain with the increase of agreement interactions. An average person could trust another one by agreeing with him/her a few times, but with some uncertainty due to the little time they know each other. With higher interactions, this uncertainty would be less with the increase of successful or failed agreement interactions. In this way, the uncertainty in the trust values should not be fixed, but change as trust is built during the game.

Based on this observation, we present a model where every node has its own *ϵ*, the parameter responsible for the degree of randomness in the choice of listener—here called exploration factor—that decays as the agent has failed communications in the game. The idea is that, in the beginning of the game, the random exploration of the minimal Naming Game holds, satisfying the need for redundancy. As the game advances, communication happens more often between nodes with a history of successes. We call this variation NG-LEF (standing for Naming Game with Local Exploration Factor—*i.e.* local *ϵ*).

Relating this local *ϵ* factor in a social scope to an uncertainty factor for *each* agent about its built trust(*w*), we produce an anthropomorphic analogy with people meeting people by chance in a social network. As time passes by, a person would trust more in others they had agreed with often and, with the decrease of their uncertainty (*ϵ*), would stop talking to people he/she knows to have disagreed with. People would then tend to talk to people that share the same opinions, giving rise to a community structure based on opinion sharing. Algorithm 3 shows the rules for NG-LEF.

In NG-LEF, a failure leads to the decay of the *ϵ* value for both nodes, meaning that nodes with higher success rates will present a slower decay of *ϵ* than for nodes that had many failures. A smaller *ϵ* means a lower randomness of this node’s listener choice. Nodes with more external edges—here called *peripheral* nodes, that exchange words frequently between communities, would have higher number of failures when comparing with nodes with less external edges, and, therefore, would have a lower value of *ϵ*. Then, peripheral nodes would choose to communicate more deterministically, often through edges with high trust, while nodes inside communities would communicate more randomly with their neighbors. So the node will behave according to its relative position in the network. This way we can have both the word dissemination aspect from the minimal NG and the restriction aspect from NG-AW, depending both on the positional role of the agent in the network and on the game step. The value of 10% for the *ϵ* decay was obtained empirically.

**Algorithm 3:** Naming Game with Local Exploration Factor

**input:** Initial value of random factor *ϵ*(0);

*ϵ*_*k*_ = *ϵ*(0) for all nodes k;

**for** *each time step:* **do**

 One agent *i* is chosen randomly to be the speaker;

 The speaker *i* chooses a listener *j* with probability proportional to

$$\begin{array}{c} p_{ij}=\frac{w_{ij}+\epsilon_{i}}{\sum_{k=neighbors}\left(w_{ik}+\epsilon_{i}\right)} \end{array}$$ (2)

where $w_{ij}=\frac{Successes_{ij}}{Attempts_{ij}}$

 **if** *the speaker’s memory is empty* **then**

  the speaker invents a new word;

 **else**

  the speaker selects randomly one from his inventory;

 **end**

 The speaker transmits the word to the listener;

 **if** *the listener has the word in his memory* **then**

  the communication is a success and both agents erase their memories keeping only the transmitted word;

 **else**

  the communication is a failure, the listener adds the word to his inventory and both agents decrease their *ϵ* in 10%

 **end**

**end**

**Fig 3:**
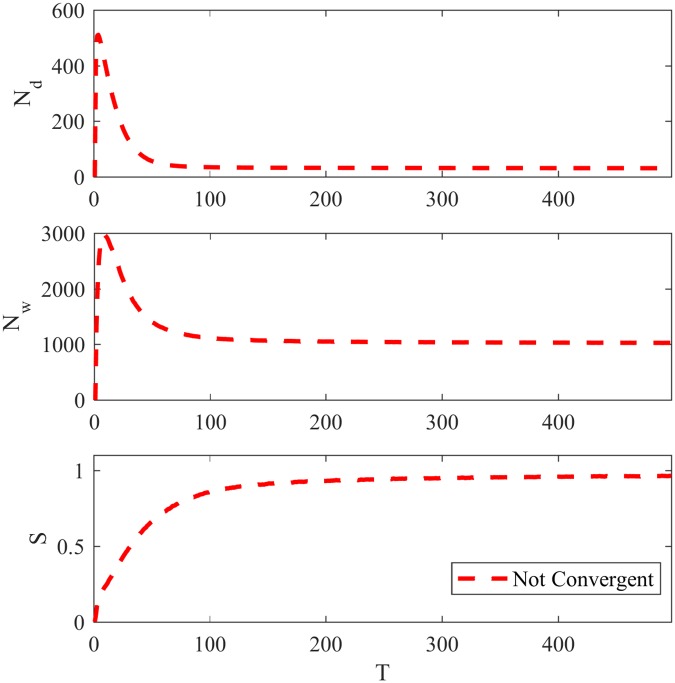
Basic features for NG-LEF. *N*_*d*_, *N*_*w*_ and *S* evolution for the Naming Game with Local Exploration Factor.

Considering NG-LEF and the same LFR network as in last subsection with *μ* = 0.3, Fig 3 displays the evolution over time of *N*_*d*_, *N*_*w*_ and *S* for the method with *ϵ*(0) = 10.

Although NG-LEF has the same behavior as NG-AW, thus permitting convergence or non-convergence in different runs with the same network and parameters, for a network with so much community structure this does not occur, as we only observed non-convergent executions. This is one of the improvements with relation to NG-AW, but it is not enough for finding communities in an arbitrary network, as we shall see in next section. For this matter, let us first introduce our last variation: the Naming Game with Secondary Memory.

#### The Naming Game with secondary memory—NG-SM

In the versions of Naming Game presented so far, agents “forget” their opinions based on a single agreement, regardless of the number of interactions the agent had using the words that were deleted. An agent can have had any number of successful communications with a word that has been present most of the time in its memory and, even so, two communications with a different word would be enough for the agent to erase completely the first word from its inventory. When comparing it to real social environment, that would be equivalent to an individual agreeing with a close group about some opinion for a considerable time, and changing it completely by talking to two people with a different opinion in a row. Differently, in real life a person may change his/her mind after a few interactions with an outsider, but would many times maintain a predisposition of believing again in a previously well agreed opinion, in which he/she believed for a long time.

It is easy to see that a NG-based model considering this aspect would restrict the entrance of external words in highly connected node groups, thus facilitating the non-convergence state that is necessary for the detection of communities. We therefore define the Naming Game with Secondary Memory (NG-SM), where each agent has a secondary inventory that keeps count of words ever heard and how many communications it had with that word. The agent will choose the word from its main memory with a probability proportional to its number of occurrences, as stored in the secondary memory. Thus, each agent will have a preference to communicate a word that had a high number of occurrences but was communicated with the agent recently. We are motivated by the social fact that one person tends to pass along an information that he/she has some conviction about. With this, we expect the peripheral nodes to still have some “confidence” in the community word even after having success with an outside word. In this way, the agent has a higher probability of communicating the community word than the outside word in the next interactions. As a matter of fact, in [22] the authors verify that the insertion of a long-term memory in agents can lead to heterogeneity in the dynamics, leading it more easily to a non-convergent meta-stable state. The proposed NG-SM model is presented in Algorithm 4.

**Algorithm 4:** NG with Secondary Memory

**input:** Initial value of random factor *ϵ*(0);

*ϵ*_*k*_ = *ϵ*(0) for all nodes k;

**for** *each time step:* **do**

 One agent *i* is chosen randomly to be the speaker;

 The speaker *i* chooses a listener *j* with probability proportional to

$$\begin{array}{c} p_{ij}=\frac{w_{ij}+\epsilon_{i}}{\sum_{k=neighbors}\left(w_{ik}+\epsilon_{i}\right)} \end{array}$$ (3)

where $w_{ij}=\frac{Successes_{ij}}{Attempts_{ij}}$

 **if** *the speaker’s memory is empty* **then**

  the speaker invents a new word;

 **else**

  the speaker selects one word *k* from his principal memory with probability proportional to the success of the word *S*_*i*_*k*__ in the secondary memory;

 **end**

 The speaker transmits the word to the listener;

 **if** *the listener has the word in his principal memory* **then**

  the communication is a success and both agents increase the number of occurrences of the transmitted word in 1 and delete all other words from the main memory;

 **else**

  the communication is a failure, the listener adds the word to his principal memory and secondary memory and both agents decrease their *ϵ* in 10%

 **end**

**end**

**Fig 4:**
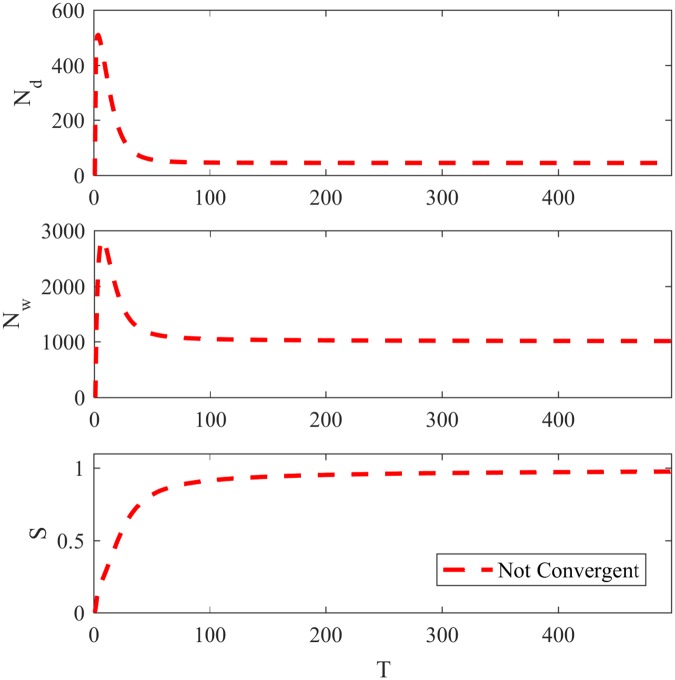
Basic features for NG-SM. *N*_*d*_, *N*_*w*_ and *S* over time in a LFR network with *μ* = 0.3 and *ϵ* = 10.

At the end of the algorithm, the agents are assigned to the community associated with a randomly chosen word from their main inventory. Fig 4 shows the evolution of *N*_*d*_, *N*_*w*_ and *S* for NG-SM for the LFR network with *μ* = 0.3, as in other subsections, and *ϵ*(0) set to 10. As in NG-LEF, NG-SM permits convergence or non-convergence in different executions, but for such network only non-convergent executions were observed.

This model, in addition to being better suited to community detection, adds another realistic social feature to the Naming Game: the preference to share an opinion proportionally to the conviction the agent has in it.

As we will see next, the addition of each of the features presented in this section progressively leads to better detection of communities.

#### Naming Game with secondary memory for overlapping community detection

The existence of multiple words in the Secondary Memory allows us to monitor the words that were more popular for each node during the game. In this way, when a node belonging to community A has a few connections to nodes in community B, its memory will change from state A (having only word A) to stage AB (having both words in its memory), and with some probability dependent on the proportion of edges towards A and towards B the memory will change to state B for a given time. So a node will “change” its community membership for a while, and this is a natural step for the proposed model. It is easy to extend this concept to nodes that belong to several different communities, that, therefore, would have many edges towards each one of them, changing states with certain frequency depending on the communities they belong to. That is the general idea behind applying NG-SM to overlapping community detection. In this way, an overlapping agent would have competing opinions present on its secondary memory, where the agent simultaneously “agrees” with different opinions. Also, interpreting NG-SM as a language game, as in the disjoint version, one can relate this behavior to people who have contact with groups that speak different languages. A person, in this situation, would use only one language at each moment, but always remembering the other ones (in the secondary memory). When speaking to people in a different language, its presence in the secondary memory will quickly be activated and the person will be using that language in next interactions.

The algorithm rules are basically the same as for disjoint community detection, the only difference being in the assignment of nodes to communities. In the overlapping case, each node can belong to a distinct number of communities, and the idea is that this number will not be given *a priori* and will not necessarily be equal for all nodes: different nodes can belong to different numbers of communities and the algorithm should determine this by itself. With this in mind, we have developed a simple post-processing step: After the game, each node *i* is assigned at least to the community referred by the word with the highest number of successes in its secondary memory. This number of successes, here called $S_{i}^{max}$, will be the basis to select the other communities agent *i* will belong to. Other high success words can be chosen as other communities if their number of successes is higher than a parcel of $S_{i}^{max}$. This parcel *p*_*l*_ is an input parameter that will indirectly limit the number of communities the node will belong to. In other words, a node *i* will be assigned to (communities related to) all words in the agent’s secondary memory that have a number of successes higher than $S_{i}^{max}*p_{l}$. If all other words from the agent’s secondary memory have a lower number of successes, the agent will belong to only one dominant community. It is easy to see that the *p*_*l*_ parameter facilitates or restricts the assignment of overlapping communities.

With a reasonable value of *p*_*l*_, this procedure will detect when two or more words were competing during the game and, in this case, assign the node to these overlapping communities. In this work we will use crisp community assignments, however the extension to fuzzy communities is trivial.

**Fig 5:**
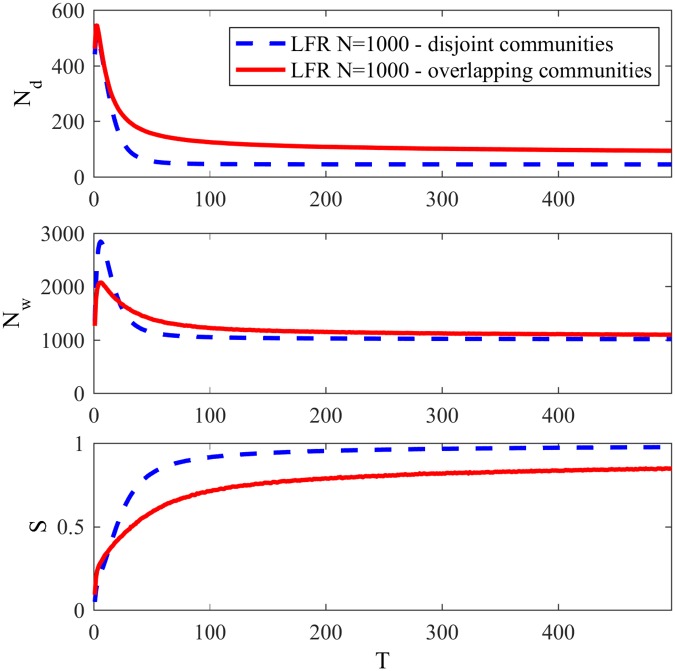
Basic features NG-SM with overlap. Number of different words, total number of words and success rate for NG-SM in LFR networks with and without overlapping communities.

In order to analyze the time evolution of the basic features—*N*_*d*_, *N*_*w*_ and *S* for NG-SM in networks with overlapping communities, we use a LFR network and set the mixing parameter *μ* as 0.1, the number of overlapping communities that each node can belong to as *O*_*m*_ = 3, the number of overlapping nodes *O*_*n*_ as 10% of N, N = 1000, the average degree as 〈*k*〉 = 10 (that is of the same order as most large real-world social networks [23]), and the rest of the parameters similar to last section: *τ*_1_ = 2, *τ*_2_ = 1, *k*_*max*_ = 50, *C*_*min*_ = 10 and *C*_*max*_ = 50. Fig 5 shows the variation of *N*_*d*_, *N*_*w*_ and *S* in time for NG-SM with *ϵ* = 10. The basic features of a LFR network without overlap with the same LFR parameters are showed as dotted blue lines, for comparison.

As we can see, there is a big difference on the behavior of the system when there are overlapping communities. There is a decrease in the number of successful communications, because in this network internal edges will link a node to more than one community, so the node would receive different words from links with high confidence, having more failed communications when those are intercalated. Another aspect that is noticeable is the lower peak of *N*_*w*_, that means that words invented were less spread through the network. This difference is not seen in *N*_*d*_ though, meaning that words are being invented but are deleted quickly. That can be observed by the steep increase of *S* in the first steps of the game.

That is an effect from the overlapping structure itself: in disjoint networks, communities are joined together by one or more *edges*, and the words invented for agents of one community tend to spread inside the community, given the larger density of connections. However, in overlapping networks, communities are joined by one or more edges *and/or* one or more *nodes*. In this way, words invented by members of one community will be spread through internal edges to other communities. When they reach a node with multiple membership, the node will easily spread them to the other communities it belongs to, making successes easier in the beginning of the game. We can also observe that the final values of *N*_*d*_ and *N*_*w*_ are significantly higher. A higher *N*_*w*_ would be expected, as nodes in multiple communities would rarely have only one word in their primary memory. As observed, approximately 10% of edges have two words in their memories, matching the parameter of the LFR network *O*_*n*_ = 10%.

## Results

### Self-organization of edges, nodes and communities with NG-based models

**Fig 6:**
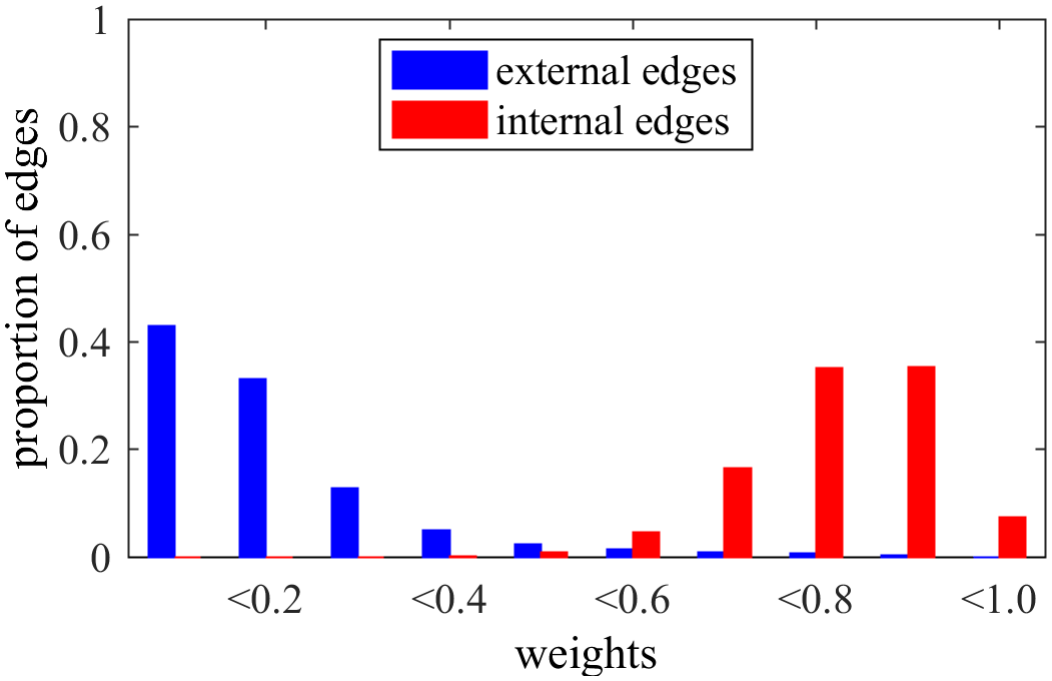
Weights NG-AW. Distribution of edges weights after playing NG-AW.

When applying the NG-based methods in LFR networks, we can observe interesting behaviors for edges and nodes, besides the detection of communities. In fact, Fig 6 shows the weights of edges after NG-AW for a LFR network with N = 1000, 〈*k*〉 = 20, *τ*_1_ = 2, *τ*_2_ = 1, *k*_*max*_ = 50, size of communities *C*_*min*_ = 10, *C*_*max*_ = 50 and *μ* = 0.2, as in last section. It is possible to observe that the weights tend to be either close to 0 or 1, i.e. the algorithm can either reinforce or weaken the edges. It does that organizing the edges: the majority of external connections have trust values close to 0, and the majority of internal edges have trust values close to 1, although they all started as 0. With this, we can see that trust builds up inside communities sharing the same opinion, as we observe in real social relationships. More specifically, we note that edges with low weights are external with high probability, suggesting a recurrent pruning of low weighted edges as an interesting possibility for detecting communities.

**Fig 7:**
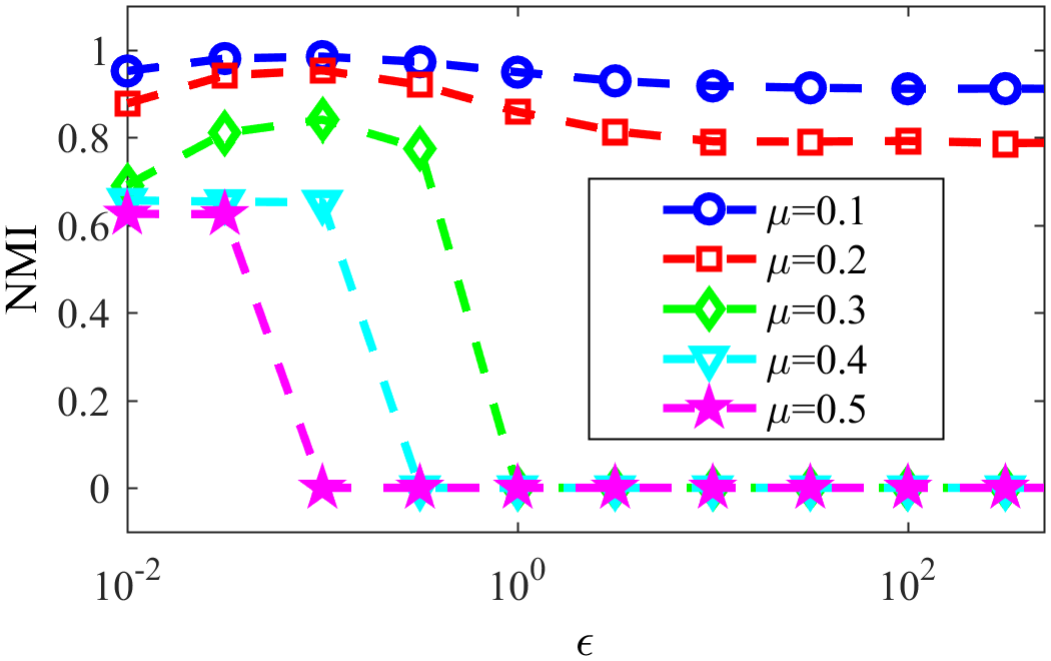
NMI NG-AW. NMI after NG-AW for LFR networks.

**Fig 8:**
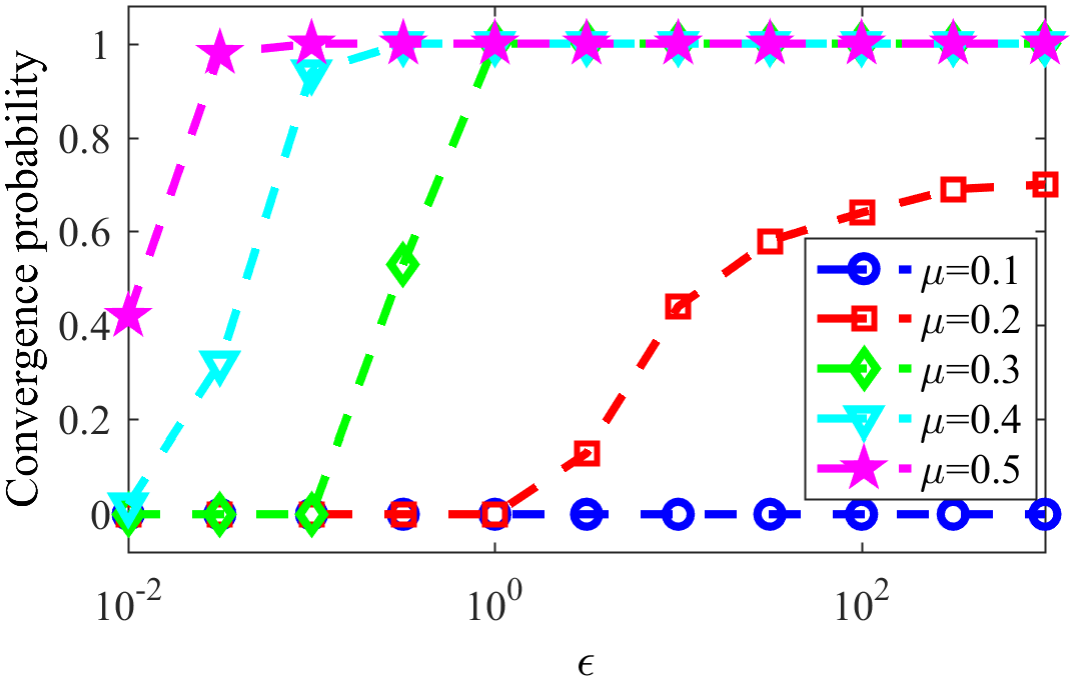
Convergences NG-AW. Probability of convergence with NG adaptive weights.

In late stages of the game, most nodes will have a single word in their memories, such words being tags for the assigned communities. Fig 7 shows the resulting NMI for LFR networks with the same parameters as in last paragraph varying the parameter *μ*, responsible for the community structure of the network, still for NG-AW. As communications happen more randomly (*ϵ* increases) and the communities in networks are less evident (*μ* increases), convergence is more likely, as seen in Fig 8.

Nonetheless, the aforementioned results suggest that model for opinion dynamics, such as the Naming Game or variations, can be adapted as a dynamical CD algorithm, uncovering the communities in an emergent way through pairwise interactions.

**Fig 9:**
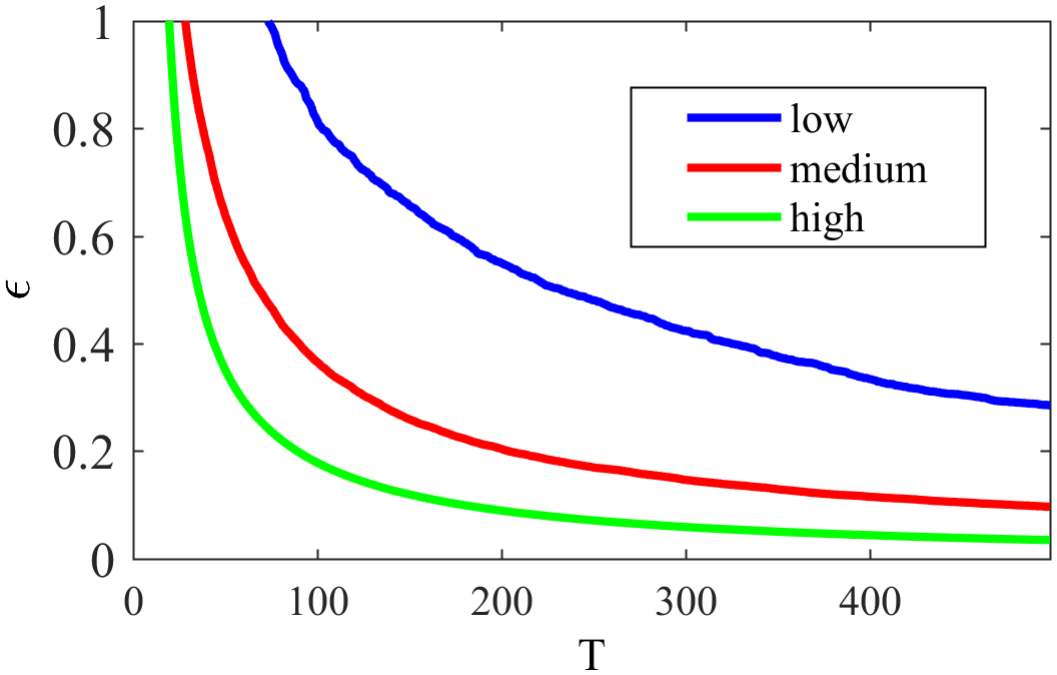
*ϵ* NG-LEF. Evolution of *ϵ* for nodes in different positions and NG-LEF—zoomed at 10% of *ϵ*(0).

In order to similarly analyze NG-LEF, we focus on two parameters: *ϵ*_*i*_ for each node *i* and the weight *w*_*ij*_ for each pair (*i*, *j*) of nodes. To verify the variation of *ϵ*_*i*_ relative to the node positioning in the network, we divided the set of nodes into 3 classes: *high*—nodes with high proportion of external edges; *medium*—nodes with medium proportion of external edges; and *low*—nodes with low proportion of external edges. The classes were set by taking into consideration the maximum and minimum proportion of external edges of the nodes in the network and dividing the interval in three. Fig 9 shows the behavior of *high*, *medium* and *low* nodes regarding the variation of *ϵ* (averages of *ϵ*_*i*_ for different types of nodes) along time, considering *ϵ*_*i*_(0) = 10, ∀*i*. For better visualization, the plot is zoomed at 10 percent of *ϵ*(0), as it is typically a small value that decays very fast.

As every node *i* has its *ϵ*_*i*_ value, the individual decrease of *ϵ*_*i*_ according to the node failure rate makes different nodes have different behaviors: the more peripheral nodes behaving more deterministically, and the more internal ones more randomly. This self-organization induced by the algorithm is maintained for larger values of *ϵ*(0) as the decrease in *ϵ*_*i*_ for peripheral nodes tend to be faster than for internal ones. These nodes, then, will choose to communicate less randomly as they have lower *ϵ*_*i*_—that is, less uncertainty. So, with less inter-community communications the nodes have more successes, tend to converge less and to maintain a single word in their memories, explaining an almost uniformly better performance compared to NG-AW.

**Fig 10:**
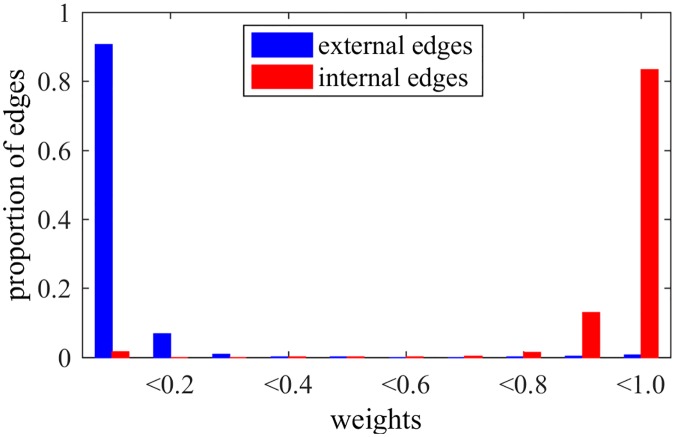
Weights NG-LEF. Distribution of edges weights after playing NG-LEF.

Still comparing NG-LEF to NG-AW, one can notice that it is more precise when classifying the edges as internal or external by their weights, as shown by Fig 10. In this way, the method outputs not only the resulting partition but also a separation of edges as external and internal (by the weights) and a classification of nodes by their relative position inside the communities (*ϵ*).

**Fig 11:**
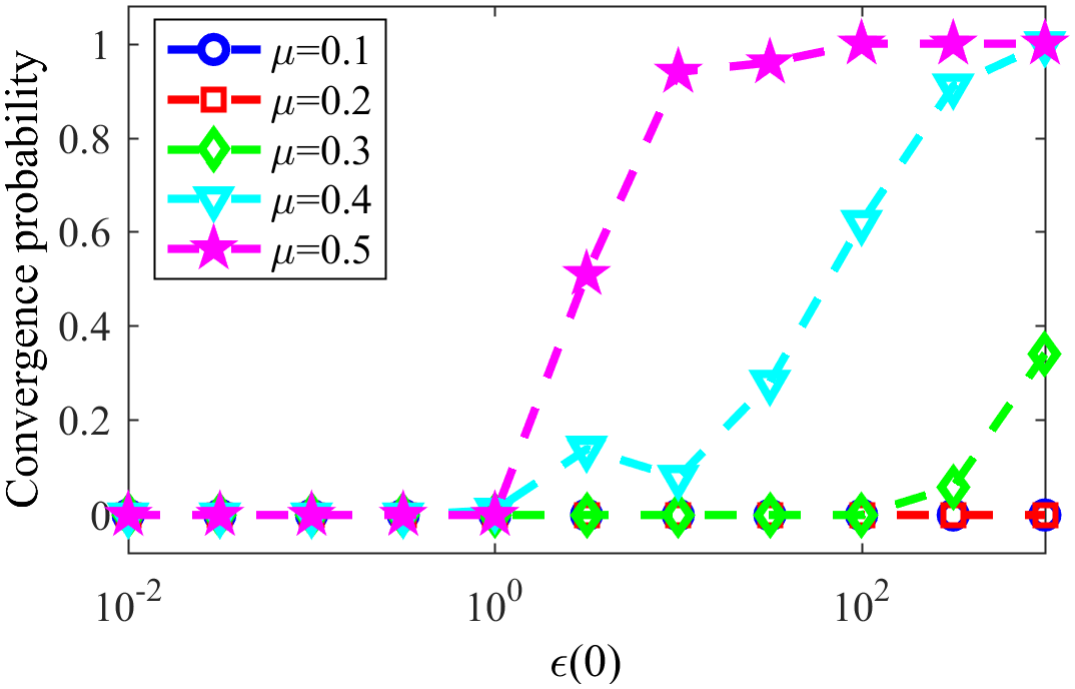
Convergences NG-LEF. Convergence probability for NG-LEF in LFR networks with different *μ* values.

**Fig 12:**
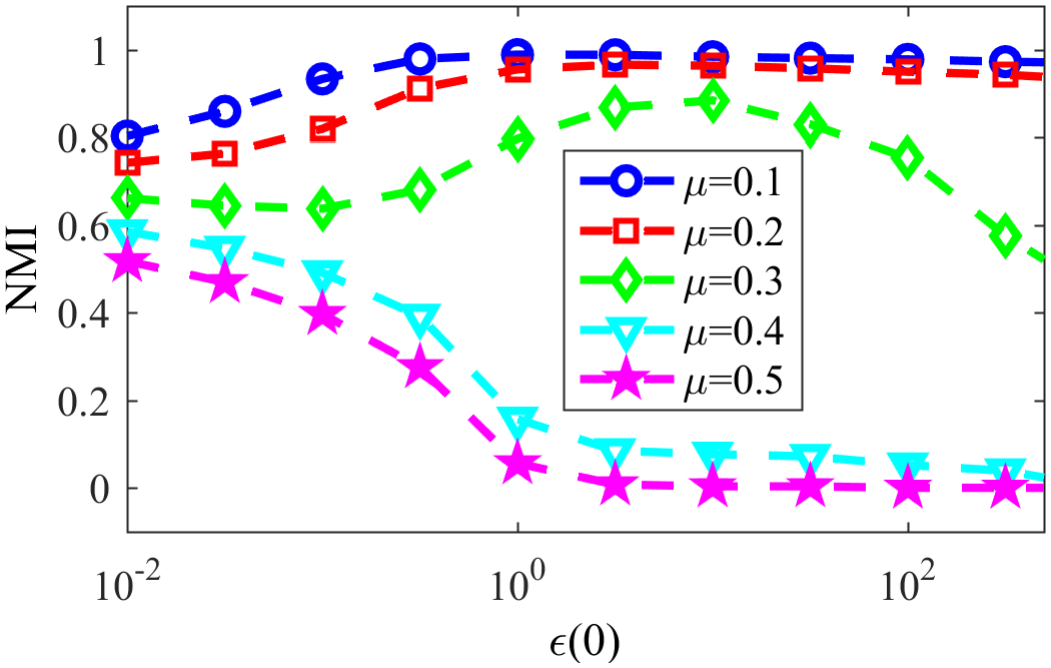
NMI NG-LEF. NMI values after NG-LEF in different LFR networks.

Fig 11 shows the convergence probability for networks generated using the LFR benchmark, for NG-LEF. Comparing Figs 11 and 8, one can notice that the convergence rate curve for NG-AW is steeper than for NG-LEF, meaning that the convergence probability grows faster for NG-AW as the input parameter increases, therefore making NG-LEF more suitable for CD purposes. To verify this, Fig 12 shows the Normalized Mutual Information obtained at the end of non-convergent games.

For networks with small and medium values of *ϵ* the classification of nodes in communities is poor, due to the reinforcement of external edges. Given the lower overall proportion of internal links, there is a slightly higher chance of external connections to be chosen for hosting the interaction. In this way, more external edges are naturally reinforced in early steps of the game, after two communications with the same randomly chosen word. With this, paths of high weight between different communities are created, making it easier for a competing word to reach a community.

If the outside word enters the community many times through different external links, the lower density of edges inside the community decreases the chance of eliminating it after a few interactions and, therefore, helps the game to reach convergence more often than in a network with lower *μ*. Also, in non convergent executions, for such networks *N*_*d*_ at the end of the game is lower than the actual number of communities (not shown), indicating that many times two communities are classified as one.

In conclusion, for NG-LEF a little increase in the mixing parameter causes a big change in the community detection accuracy, and generates a superior limit to the input value *ϵ*(0), from which the game always reaches convergence for networks with low community structure.

**Fig 13:**
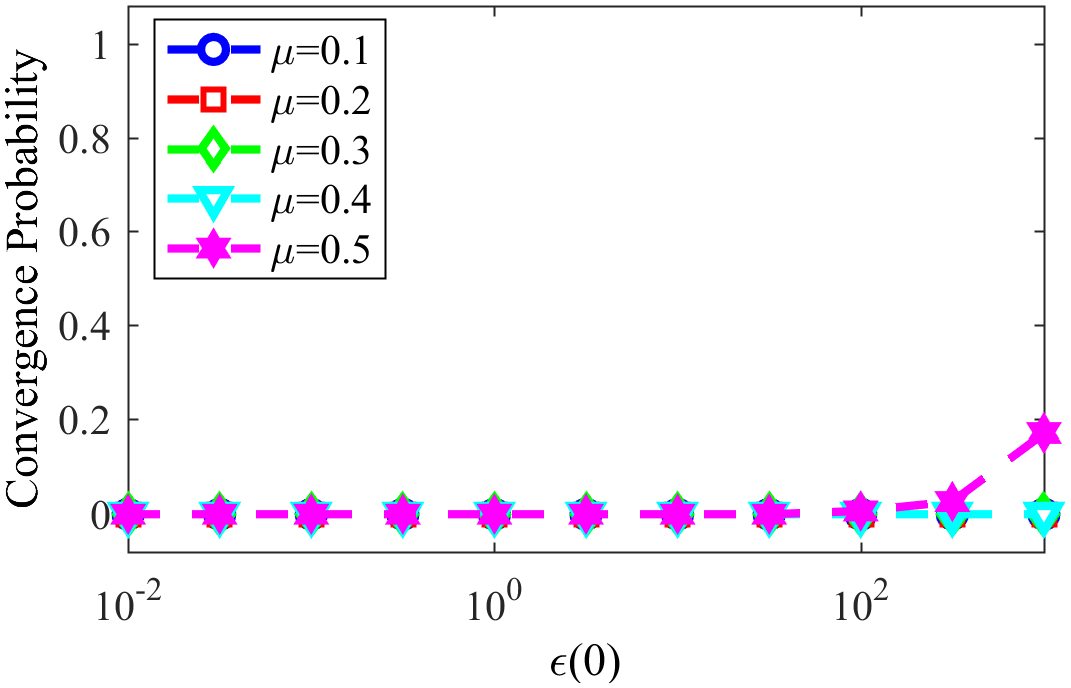
Convergences NG-SM. Convergence probability for NG-SM for various LFR networks.

Based on that conclusion, we finally analyze NG-SM: Fig 13 shows the convergence probability of NG-SM played among agents on a topology of the same LFR networks. Comparing it to Fig 11, we can see a large improvement for NG-SM for all networks, especially for those with lower community structure.

**Fig 14:**
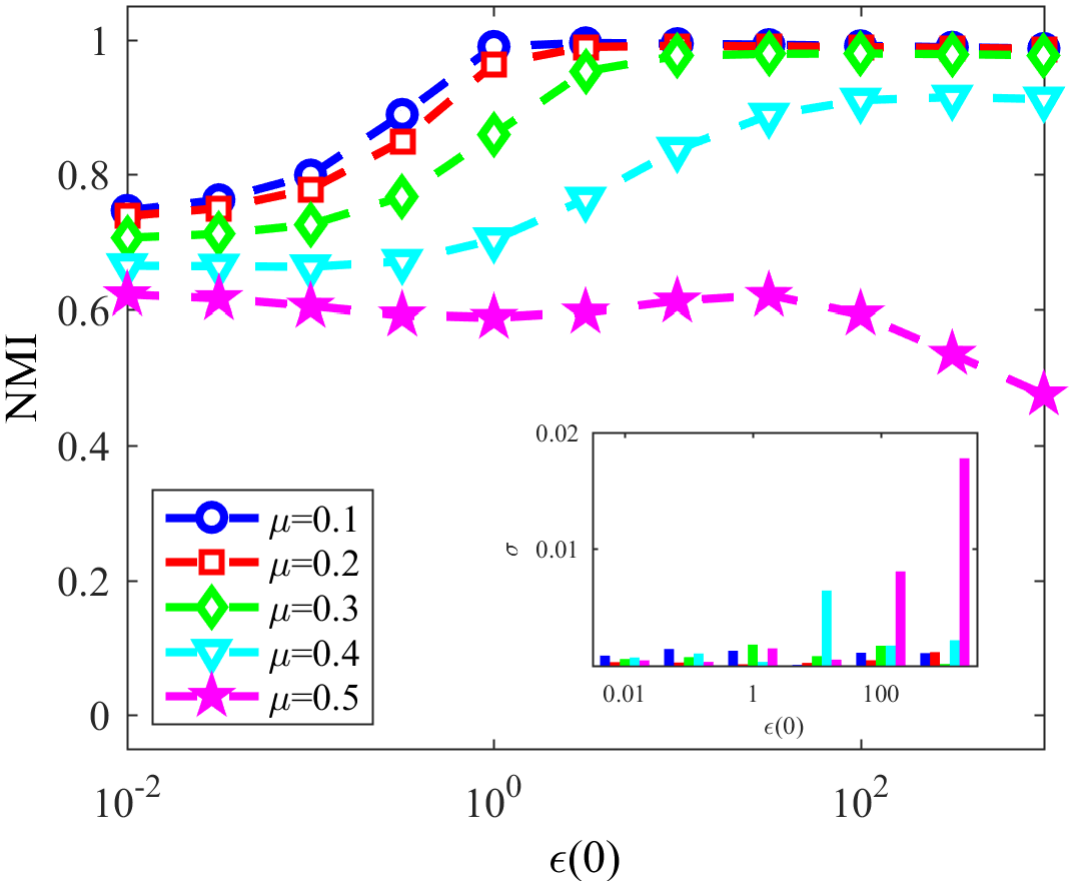
NMI NG-SM. Resulting NMI for NG-SM in LFR networks. Detail: Standard deviation of the obtained NMI.

Now let us consider the actual community detection accuracy, showed in Fig 14, and compare it with Fig 12. Networks with low community structure, like those with *μ* = 0.4 and *μ* = 0.5, that were intractable with NG-LEF—having low NMI in the rare non-convergent executions—now reached NMI ≈ 0.9 and 0.6 respectively, with high probability of non-convergence. NG-SM maintains the stable behavior of networks with stronger community structure as *ϵ*(0) gets higher, and presents a plateau of smaller height for networks with less community structure, presenting altogether a better overall community detection accuracy than NG-AW and LEF. In the detail we show the standard deviation of NMI for the same networks and *ϵ*(0) values. Its noticeable that the numeric value of the standard deviation is small, showing that different executions have similar behaviors. This value tends to get higher as the mixing parameter of the network increases. However, the highest value of the standard deviation is lower than 0.02.

Table 1 shows the resulting number of communities for Naming Game MS and different *ϵ*(0) values. As can be seen, for higher values of *ϵ*(0) we have a number close to the actual number of communities. This number gets smaller as the mixing parameter increases, meaning that separate communities are being tagged as one, explaining the lower overall NMI for such networks.

**Table 1:** Number of communities obtained by NG-SM versus original number of communities.

	Number of communities						
	Original	*ϵ*(0) = 0.1	*ϵ*(0) = 1	*ϵ*(0) = 10	*ϵ*(0) = 100	*ϵ*(0) = 1000	*ϵ*(0) = 10000
μ = 0.1	36	167,68	37,86	35,83	35,87	35,88	35,84
μ = 0.2	44	180,09	51,57	43,37	43,19	43,29	43,12
μ = 0.3	46	194,24	81,05	44,97	43,8	43,48	43,55
μ = 0.4	44	207,94	112,61	55,22	39,78	36,81	35,77
μ = 0.5	41	215,97	124,15	72,88	34,06	18,85	14,34

**Fig 15:**
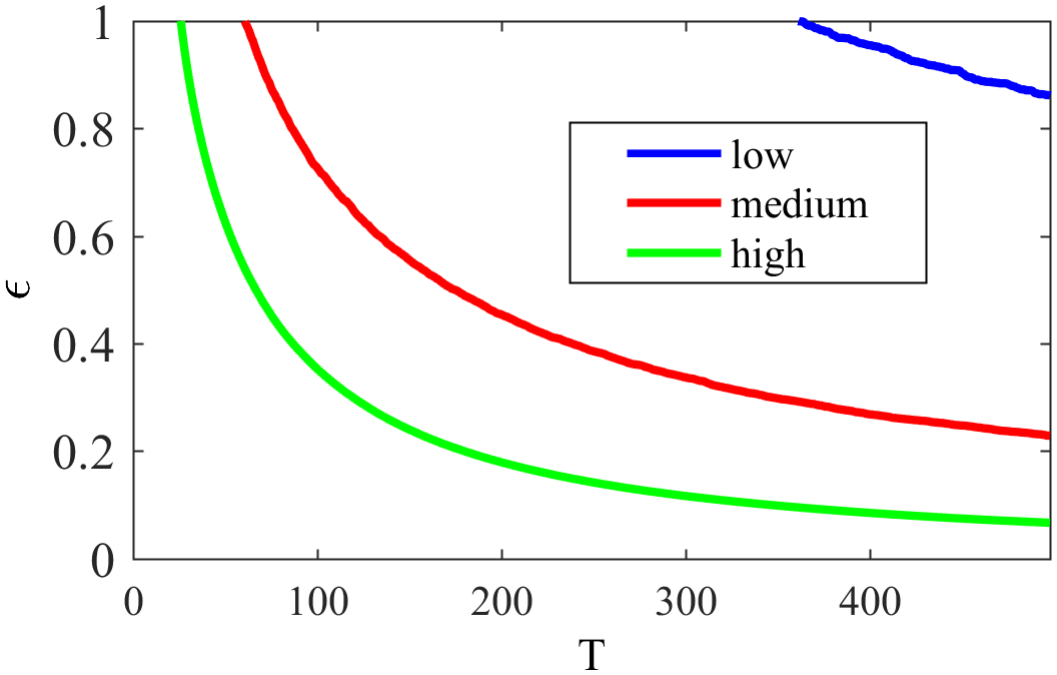
*ϵ* NG-SM. *ϵ* evolution for nodes in different positions and NG-SM—zoomed at 10% of *ϵ*(0).

**Fig 16:**
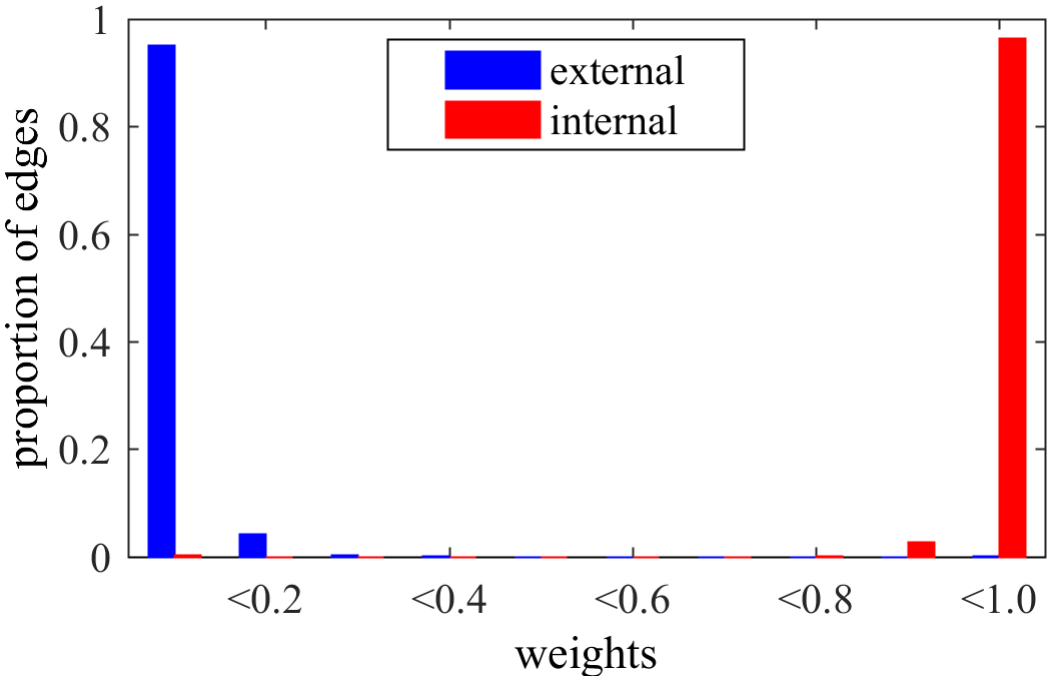
Weights NG-SM. Distribution of edges weights after a NG-SM execution.

Another point to be analyzed in NG-SM is one of the key aspects of NG-LEF: its ability to separate nodes and classify edges. Trying to verify if NG-SM maintains such quality, Figs 15 and 16 show the *ϵ* evolution and the histogram of edge weights with the same *μ* = 0.2 LFR network, and *ϵ*(0) = 10.

Analyzing the *ϵ* evolution, in comparison with Fig 9, NG-SM not only maintains the classification of nodes by their *ϵ* values, but we observe throughout the game a larger numeric difference between classes averages. This indicates that the difference in the number of failures for different nodes is larger. Moreover, the values for all classes are larger overall, indicating more uncertainty in the opinion sharing process, without reaching consensus. This is important, as the random aspect of the communications is instrumental for gathering intrinsic information about the topology.

Looking at the final weights of edges and comparing it to Fig 10, for external edges we see basically the same: almost all edges have weights close to 0. For internal edges, however, we see a considerable improvement in the proportion of internal edges that were reinforced, having weights close to 1, meaning that more internal edges are participating in the communications, and, therefore, more edges overall actively play the game. This classification of edges in external and internal happens as the weights of edges are built, *i.e.*, with the history of successful communications. As in NG-LEF, this happens as an emergent behavior in non-convergent executions.

In short, we empirically established that NG-SM has lower convergence probability, better classification of both nodes and edges, and higher overall accuracy in detecting communities, compared to NG-LEF and NG-AW.

### Network parameters impact on NG-SM

**Fig 17:**
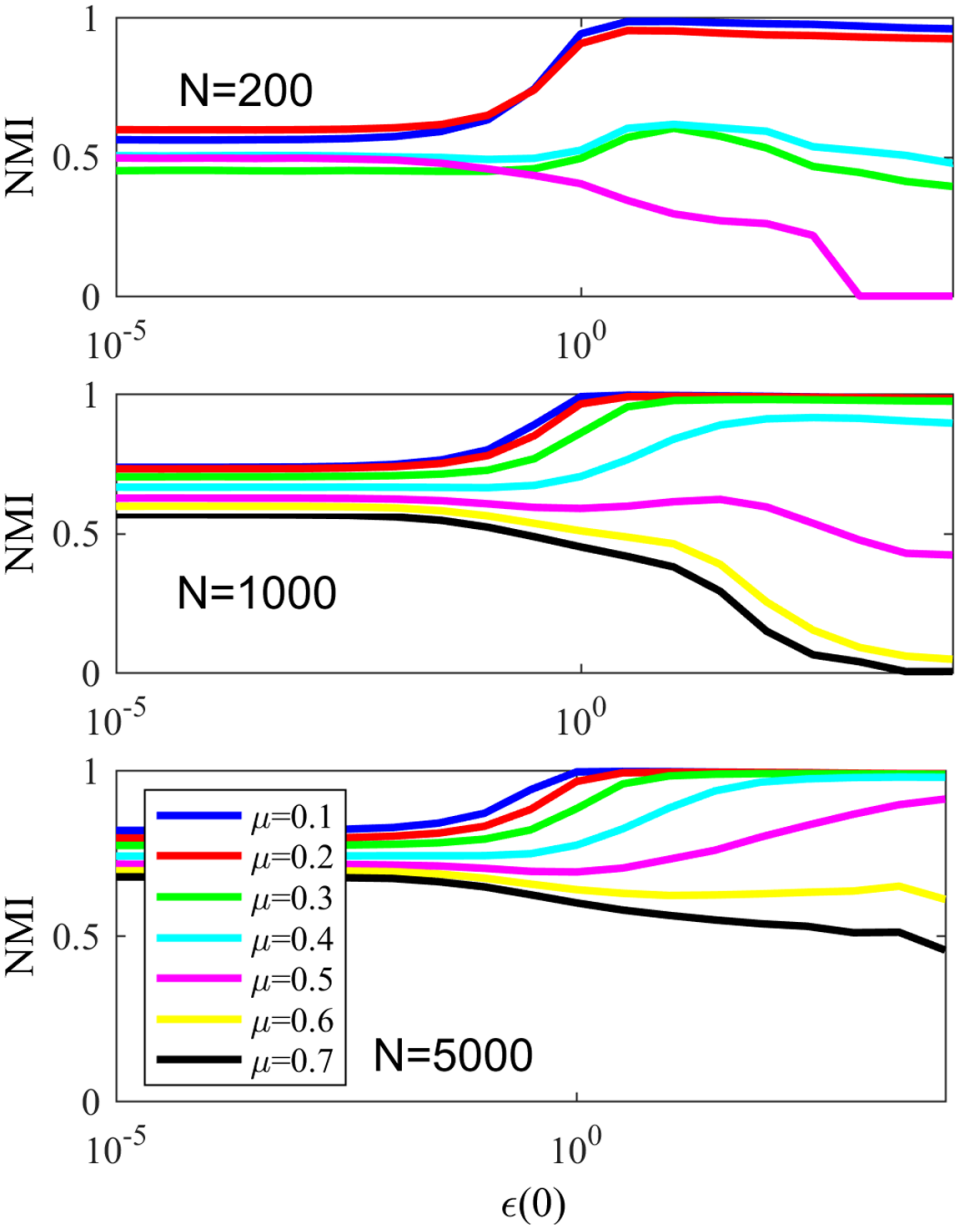
NMI varying N. Normalized Mutual Information for NG-SM with LFR networks and different sizes.

Even though for networks with configurations as in last subsection we have promising results, it is worth to analyze the influence of other network parameters for community detection. As a first step, Fig 17 shows the NMI for networks with the same parameters, varying the network size and *μ*. As we see, for a larger network, NG-SM in fact works even better. This happens because, for a word to enter and dominate a community it has to reach it from various directions through high weight paths from one community to the other, in order to obtain enough successes with the outside word to spread it. Due to the existence of more words—and therefore more competition for success—and to the fact that these paths would have larger length for a larger network (thus existing with lower probability), convergence rarely happens and words tend to get trapped in the multi-word regime more often.

This suggests that this algorithm is better suited for applications in large networks. As our aim is to study the dissemination of opinions in social populations that are usually very large, this conclusion is relevant.

**Fig 18:**
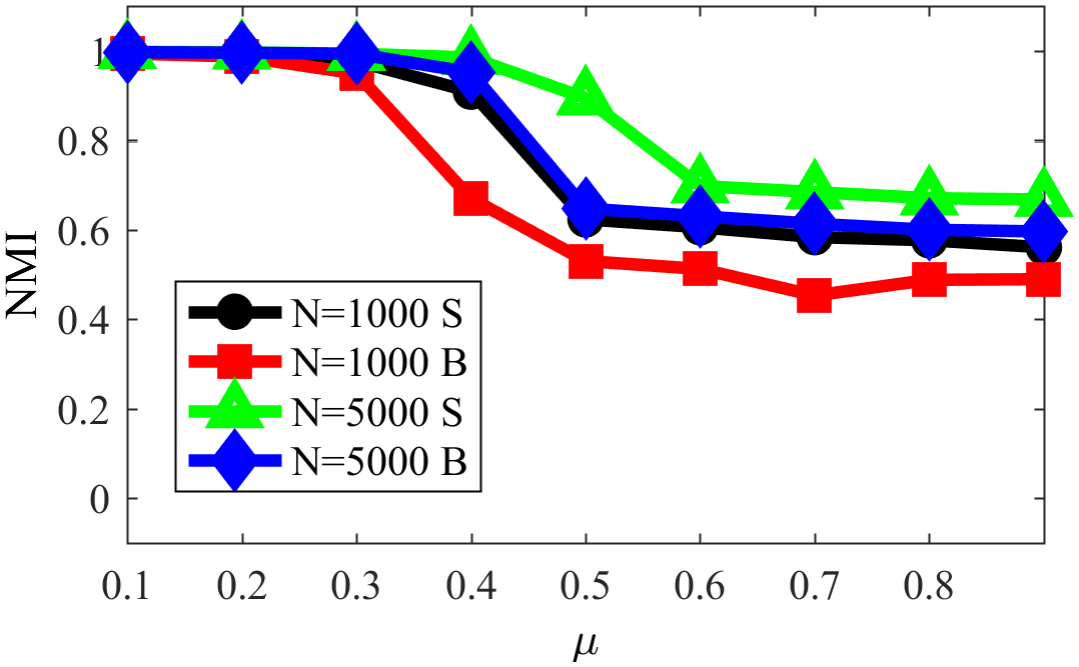
NMI for NG-SM in LFR networks with different sizes and community sizes.

**Fig 19:**
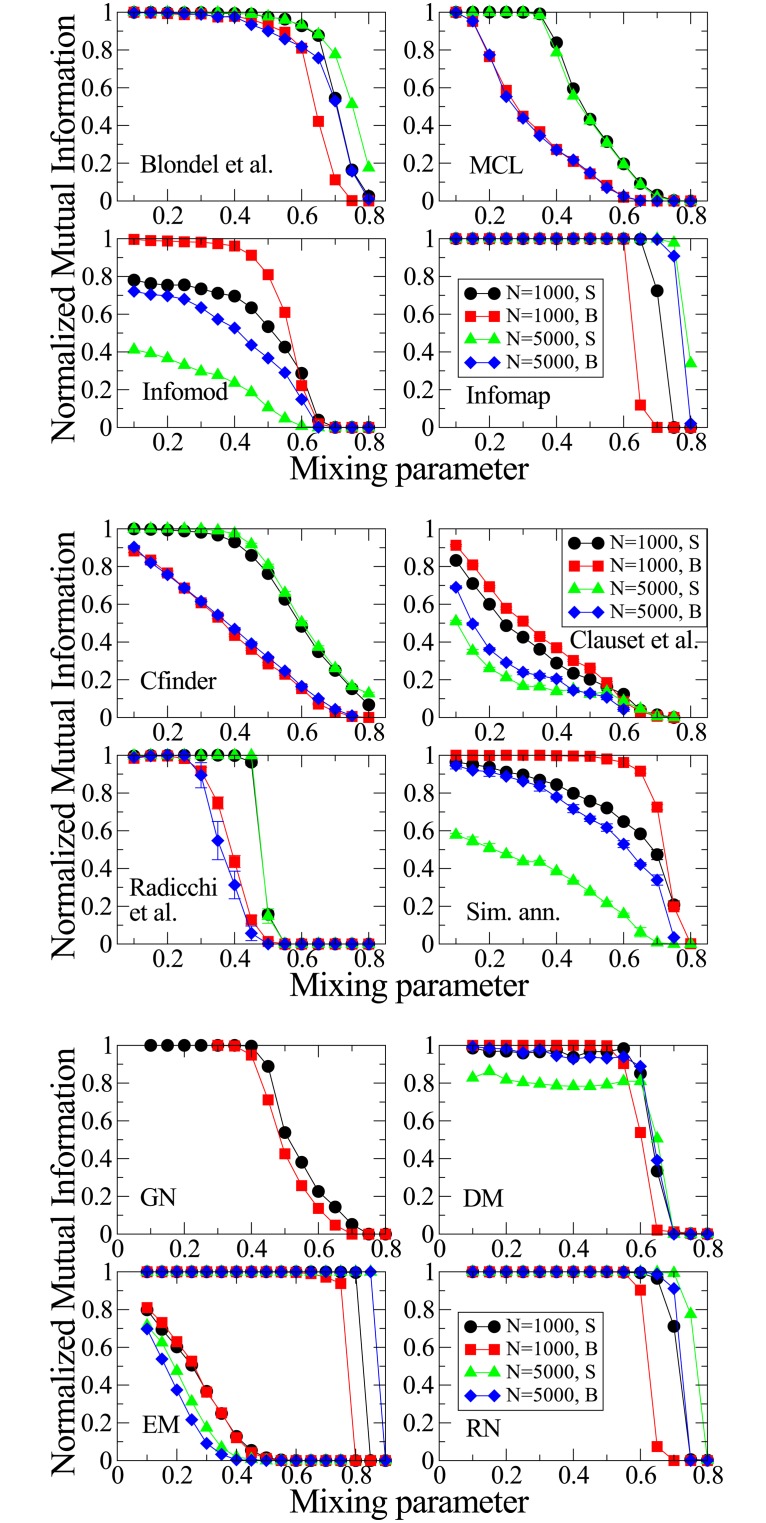
NMI for different algorithms for community detection for comparison [24]. Reprinted with permission.

In order to analyze the impact of communities size, Fig 18 presents the NMI obtained with NG-SM for networks in which we vary the network sizes and communities sizes, where “S” stands for small communities, from 10 to 50 nodes, and “B” stands for big communities, from 20 to 100 nodes. Other parameters remain the same as for the networks studied so far in this section. One could compare Fig 18 with the obtained NMI for several other Community Detection algorithms from [24], presented in Fig 19, since the networks used in Fig 18 are equivalent to those used in [24]. When doing so, we can notice that, differently from the majority of CD algorithms in [24], NG-SM has better accuracy for larger networks than for smaller ones, as we had just concluded. Also, it seems to work better when the communities in the network are smaller, again in contrast with many of those algorithms. That is interesting because dynamical algorithms tend to use only local information, building the communities from inside-out (bottom-up), where small communities are found first. Other algorithms commonly use global information, building the communities from outside-in, where larger communities are found first, explaining those behaviours and, once again, stressing that the choice of a CD algorithm should be related to the problem at hand.

Looking now at the actual NMI values, we see that, with exception of the RN and Infomap algorithms, that are better in almost all instances, our model has accuracy comparable and many times better than most community detection algorithms used for comparison in this work, specially for networks with low community structure (large *μ*).

**Fig 20:**
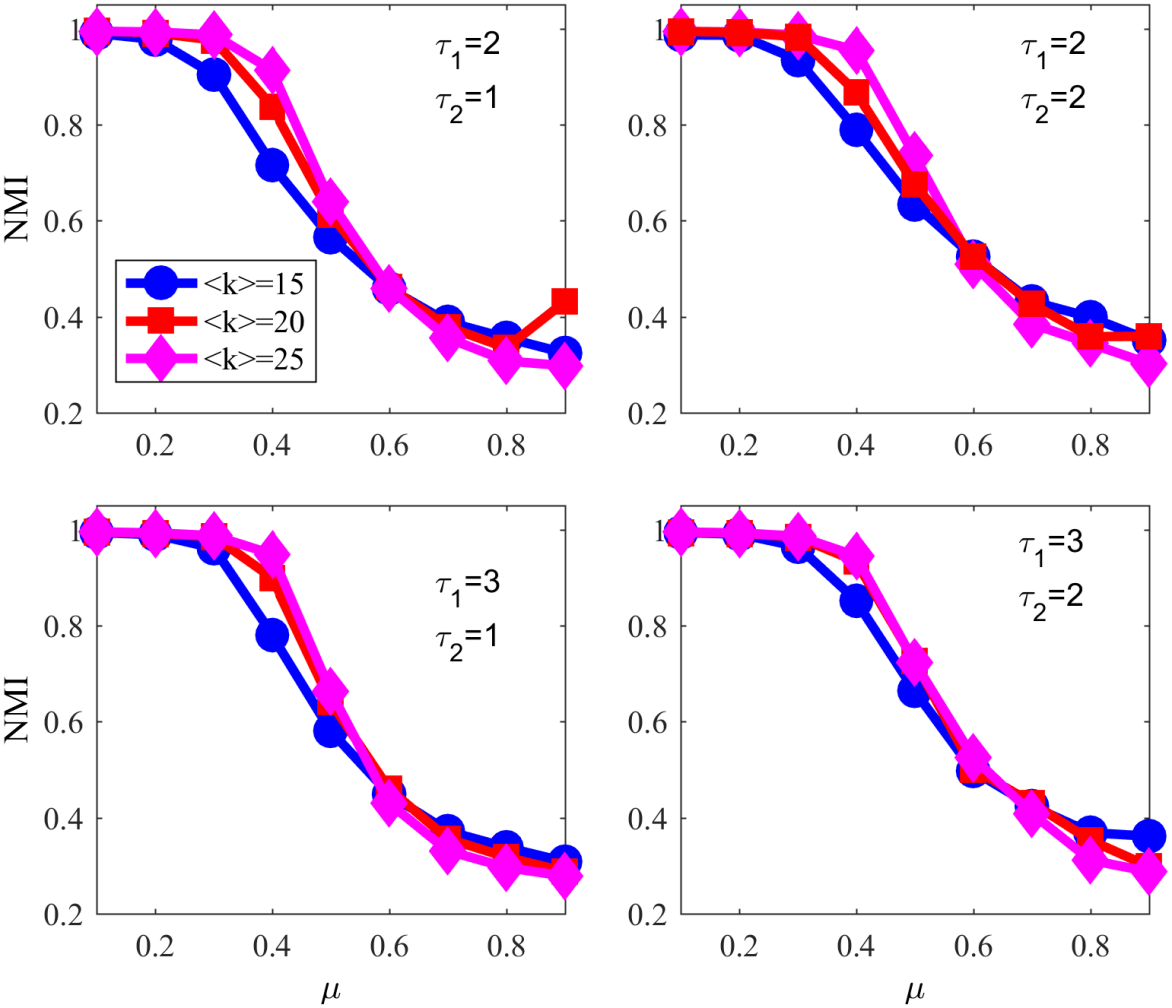
NMI varying *τ*_1_, *τ*_2_ and 〈*k*〉. NMI for NG-SM in LFR networks with 1000 nodes varying exponents and average degree.

Now, let us focus on other parameters, namely community size distribution, degree distribution and average degree; Fig 20 shows NMI values over different values of *μ* for NG-SM and NG-LEF with *ϵ*(0) = 10 and varying parameters for networks with N = 1000 nodes and small communities size—from 10 to 50 nodes— 〈*k*〉 = 15,20,25, *τ*_1_ = 2 or 3 and *τ*_2_ = 1 or 2, that are the extremes of the exponents’ ranges [15].

One thing that we can observe is that the NG-SM algorithm works better as the average degree of the network grows, for all community sizes and degree distributions. As the number of connections inside communities is larger than the number of connections between communities, to increase the average degree creates more internal connections than external ones, reinforcing the community structure.

About the degree distribution, with a smaller exponent, *i.e.* a wider distribution of degrees, we have worse performance in detecting communities: for all networks *NMI*_*τ*_1_ = 3_ > *NMI*_*τ*_1_ = 2_. The presence of hubs facilitates the spread of an outside word in a community: once the hub has the outside word with a relative high number of successes, it will spread it to many different agents that will communicate among themselves and increase that word success rate in their secondary memories.

The same behavior happens with the community distribution exponent: *NMI*_*τ*_2_ = 2_ > *NMI*_*τ*_2_ = 1_ for all networks tested, meaning that the algorithm detects communities of similar sizes better. On the other hand, we see that the behavior in detecting communities is robust when changing LFR parameter values, even though it slightly changes from case to case.

### NG-SM behaviour in real world networks

As mentioned earlier, our aim is to show how communities can emerge naturally in a simulated social environment, so it would be interesting to verify the behaviour of NG-SM and NG-LEF in real world networks. For this, we calculate the modularity values for the partitions obtained at the end of the NG-based games.

The real networks used in our work are: *Dolphins*—the network of communications among dolphins [25, 26]; *Football*—Network of American football games [14]; *SFI*—Santa Fe Institute scientists collaboration network [1]. *E-mail*—An university network of e-mail communication [27]; and *Power Grid*—The Western States power grid of the United States [28]. Even though some of these networks can exhibit weighted or directed edges, in this work we consider all edges unweighted and bidirectional.

Table 2 details the size and modularity values for the real networks. This modularity values were obtained from [29], using the Label Propagation Algorithm [30].

**Table 2:** Real networks used in this work and their modularity values from LPA.

Network	N	Modularity LPA
Dolphins	62	0.5
Footbal	115	0.6
Santa Fe	118	0.70
Email	1133	0.28
PowerGrid	4941	0.81

**Fig 21:**
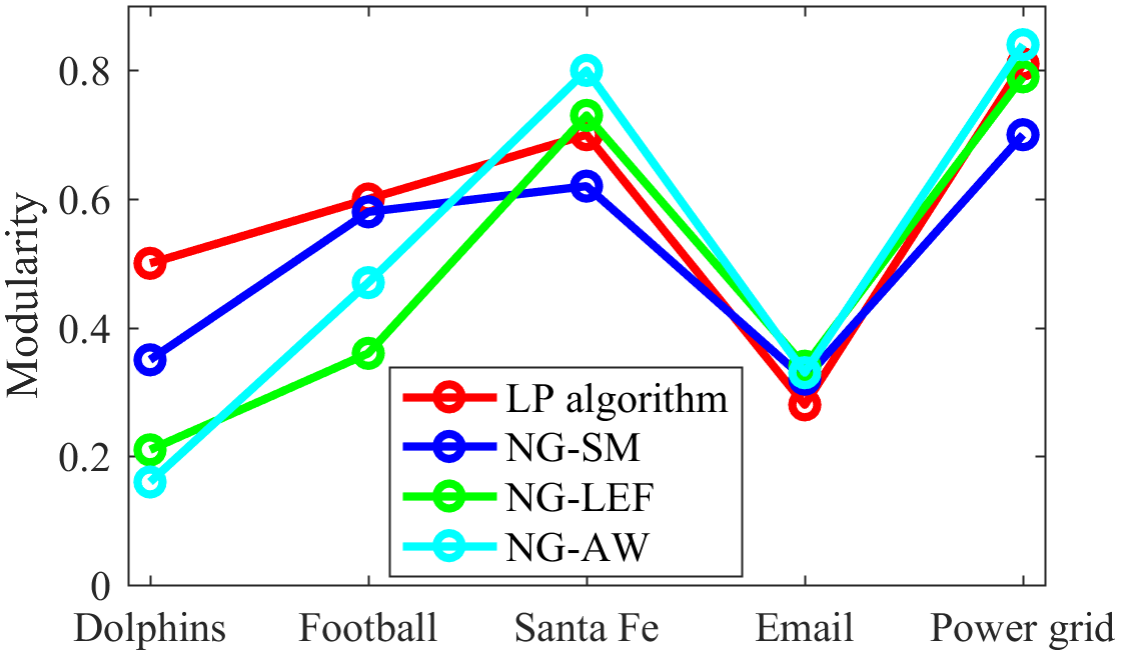
Modularity values. Modularity values obtained for five different real networks.

Fig 21 presents the modularity values obtained after the games NG-AW, LEF and SM in comparison with the modularity obtained in [29] for the aforementioned networks. The input values were *ϵ* = 0.01, *ϵ*_0_ = 0.01 and *ϵ*_0_ = 100, respectively.

We can observe that, overall, the three variations of the NG produce values close to those obtained with other CD algorithms. As mentioned earlier in the paper, the NG-based algorithms present better performances in larger networks, when compared to smaller ones. That can be seen in Fig 21, where there is a larger difference among the values of modularity when the network is small. This difference becomes lower when the size of the network increases.

Next we exploit a branch that is growing fast in the Community Detection literature: Detection of Overlapping Communities.

### Overlapping community detection with NG-SM

**Fig 22:**
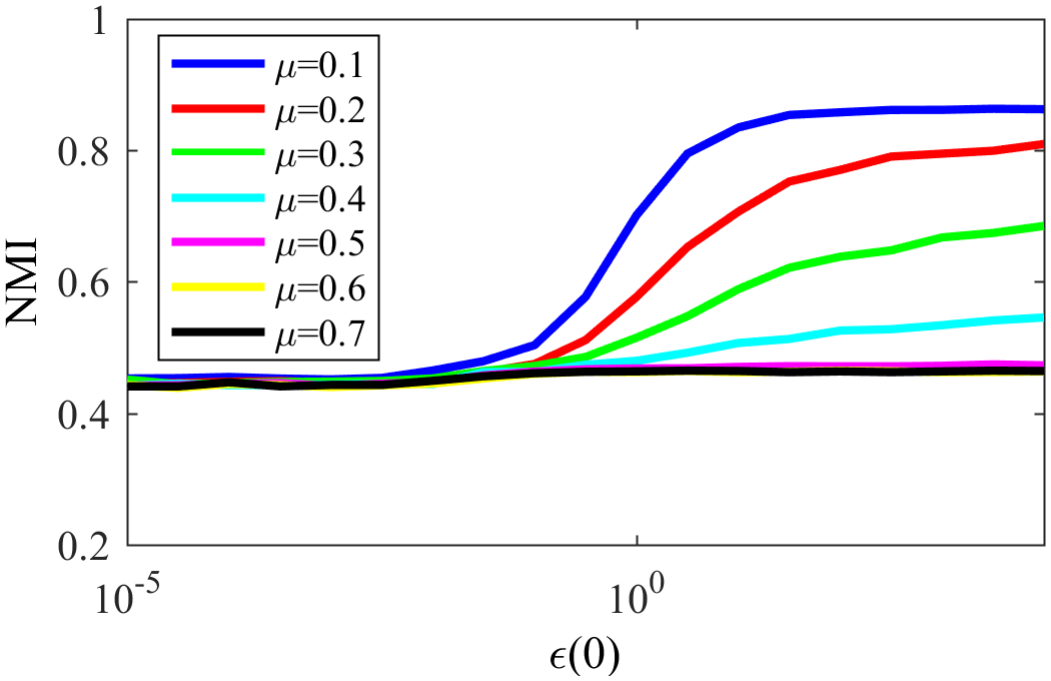
NMI overlap. NMI for NG-SM in LFR networks with overlapping communities.

We have seen that the NG-based algorithm incorporating the three social features results in the emergence of the existing communities for artificially generated networks and real world networks, in a natural way. In this subsection, we analyze the results for networks with overlapping communities. We consider overlapping LFR networks with *O*_*m*_ = 3, *O*_*n*_ as 10% of N, N = 1000, 〈*k*〉 = 10, *τ*_1_ = 2, *τ*_2_ = 1, *k*_*max*_ = 50, *C*_*min*_ = 10 and *C*_*max*_ = 50. Fig 22 shows the NMI (overlap version) [13] for LFR networks with different *μ* values, for different values of *ϵ*(0) and *p*_*l*_ = 0.9.

As in the disjoint case, when we increase *μ*, the NMI value decreases. However, as expected, the NMI value is lower than for the disjoint case, for every network. This can be explained by the decrease on the average degree 〈*k*〉, that, as we saw in last section, has a negative influence on the NG-SM accuracy. Another thing that can influence is the actual multi-membership in the game: when a node belongs to two communities A and B, it will periodically receive both words *a* and *b* resulting from interactions with each community, respectively; This results in many unsuccessful interactions with members of its own communities, thus weakening the weights of these internal edges—that should be high—and making more probable for external edges to be chosen to host a communication. Here, an external edge will be an edge linking two nodes that share no membership, while all other edges are considered internal ones.

When internal edges and external ones have similar weights, the choice of listener is random and words that should be deleted are propagated to as many communities as the node belongs to. This fact is also the responsible for the absence of convergent executions. If a node has many connections to more than one community, the shifting from one word to the other makes the node have multiple words in its memory most of the time, as evidenced by the *N*_*w*_ evolution. Also, it is noticeable that higher values of *ϵ*(0) lead to better community detection, given the absence of convergences.

**Fig 23:**
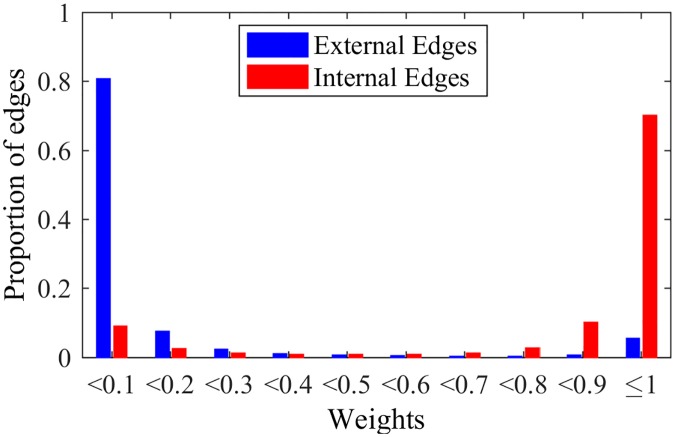
Non-overlapping weights. Histogram of weights distribution for non-overlapping edges.

**Fig 24:**
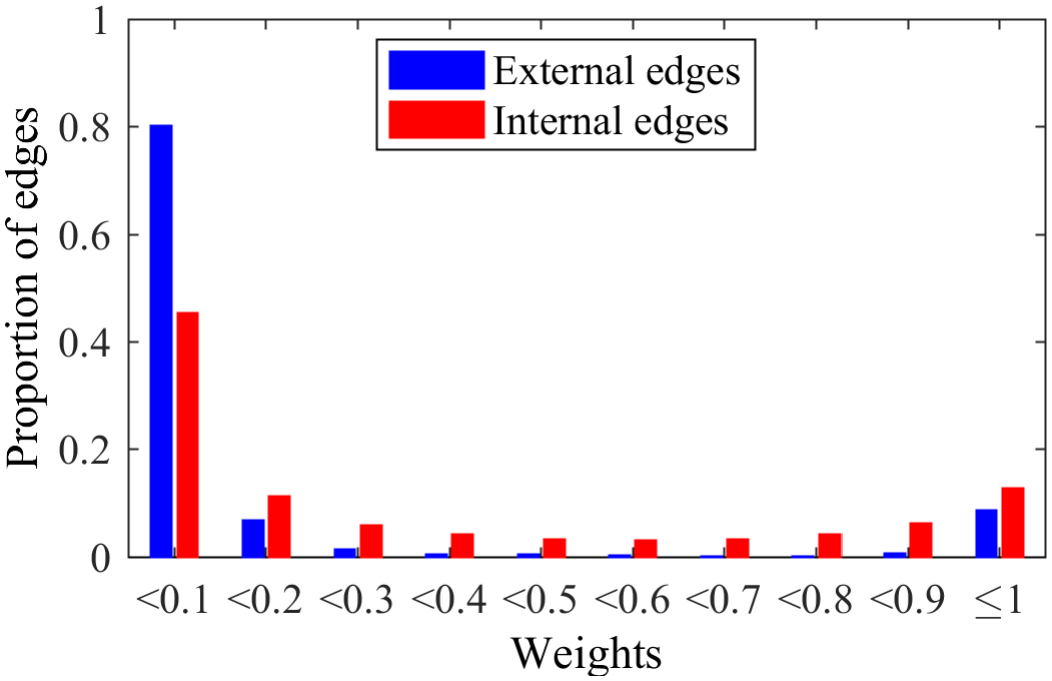
Overlapping weights. Histogram of weights distribution for overlapping edges.

In order to verify this explanation, let us look at the weights of the edges—that should have lower values even if they are internal if the node is overlapping (*i.e.*, belonging to more than one community) and for the rest of the edges should be close to 0, if they are external, and close to 1 if they are internal, like in the non-overlapping case. Figs 23 and 24 show the weights of the edges of nodes that do not overlap and do overlap, respectively. If an edge joins an overlapping node to a non-overlapping one, the edge will also be considered overlapping.

**Fig 25:**
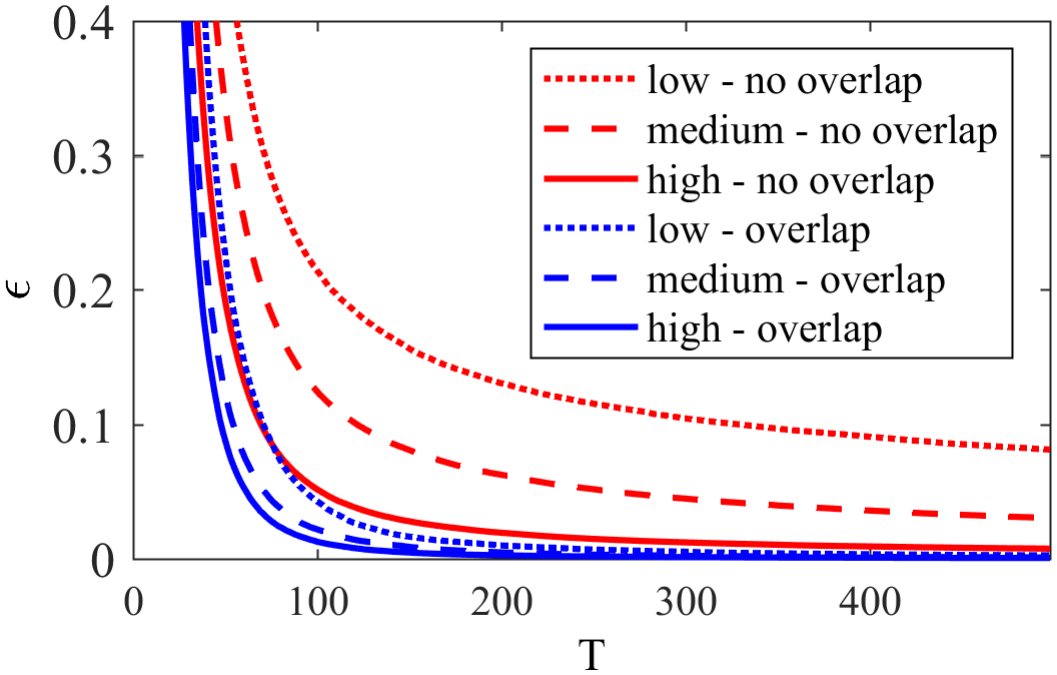
Overlap *ϵ* evolution. Time evolution of *ϵ* values for different nodes with and without overlap.

Nodes that belong to a single community have behavior similar to the disjoint case. For overlapping nodes the distribution of the external edges is also similar, being close to zero with high probability. On the other hand, the internal edges’ weights are very different: almost half of them are close to 0 and the rest of the internal weights are distributed almost uniformly between 0.1 and 1. If the weights cannot distinguish internal from external edges, for an overlapping node *i* the choice of listener *j* will be independent of the weight of edge *ij* and will happen with probability proportional to *ϵ*_*i*_ for all neighbors, *i.e.*, randomly. The value of *ϵ*_*i*_ is dependent on the initial *ϵ* and the number of failures of node *i*. As discussed, an overlapping node would frequently receive words from the different communities it belongs to, resulting in a higher failure rate and a lower *ϵ* value. As a matter of fact, Fig 25 shows the time evolution of *ϵ* values for nodes with low, medium and high proportion of external edges (overlapping nodes in blue, and non-overlapping nodes in red). The figure is zoomed for better visualization.

We can observe that *ϵ* decays faster in time for overlapping nodes, and that these values depend less on their proportion of external edges: they are similar for nodes with low, medium and high proportion of external edges. Also, one can notice that the value of *ϵ* for overlapping nodes is, most of the time, smaller than the average for nodes with the highest rate of external edges, making them behave more restrictively during the game. On the other hand, as the edges’ weights from this kind of nodes are distributed regardless of being internal or external, the chance of choosing an internal or external edge for communication will be proportional to their rates of external edges.

Even though the analysis above can seem unpromising, in the overlapping case the assignment of communities is based on the secondary memory and not in the main one. In this way, if a node have received (or had successes with) two or more words repeatedly coming from different communities it belongs to, it will probably be assigned to these communities, if the rate of occurrences of these words are similar, according to the input parameter *p*_*l*_.

**Fig 26:**
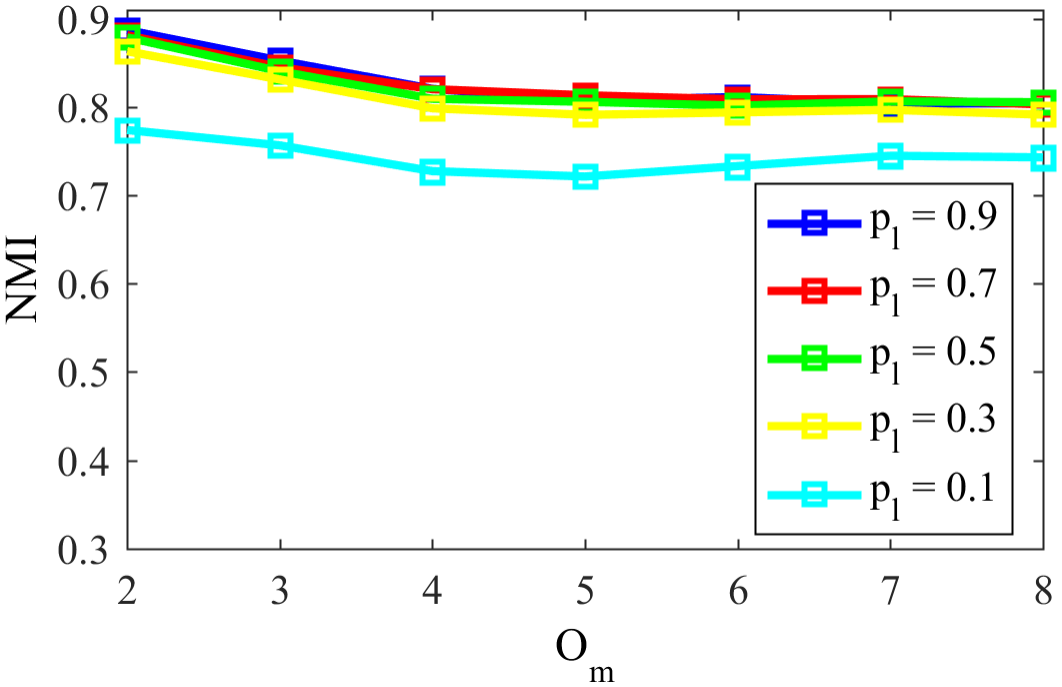
Influence of *p*_*l*_ variation. NMI for LFR networks with varying *O*_*m*_ and various *p*_*l*_ values.

In order to analyze the impact of the parameter *p*_*l*_ on the dynamics, Fig 26 shows NMI values for a LFR network with *μ* = 0.1, *O*_*n*_ = 10% of N, N = 1000, 〈*k*〉 = 10, *τ*_1_ = 2, *τ*_2_ = 1, *k*_*max*_ = 100, *C*_*min*_ = 20 and *C*_*max*_ = 50 as in [23]. The *ϵ*(0) is set as 10^5^ and the number of communities a node can belong to varies from *O*_*m*_ = 2 to *O*_*m*_ = 8.

As we see, only for very low *p*_*l*_ (*p*_*l*_ = 0.1) we have significant difference on the performance in community detection, uniformly decreasing the resulting NMI in approximately 0.1 for all values of *O*_*m*_. A *p*_*l*_ of 0.1 would mean assigning to a node a second community related to a word that had only 10% of the occurrences of the dominant word. The second word, in such cases, does not necessarily represent a competing word.

On the other hand, for larger values of *p*_*l*_ we have uniformly a better performance. That happens because if the node belongs to *O*_*m*_ different communities, in late stages of the game it would have received the *O*_*m*_ different words many more times than other received random words that should appear just a few times, before they are eliminated. For the same reason, the NMI value decays slightly with the first increases in the number of overlapping communities *O*_*m*_ and is stable for larger *O*_*m*_, also being robust regarding the number of communities the node can belong to.

**Fig 27:**
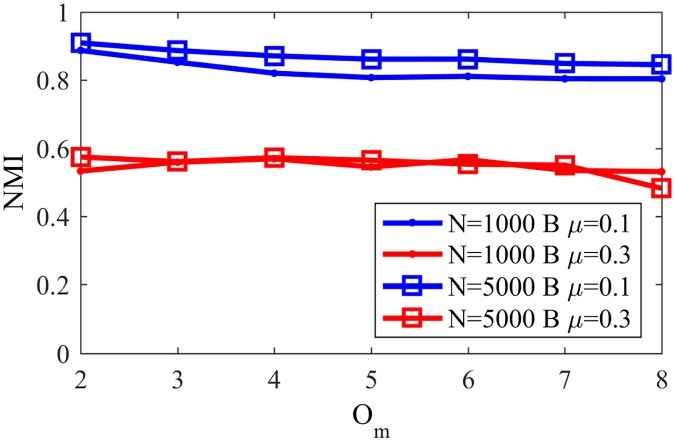
NMI overlap with big communities. NMI in LFR networks with big overlapping communities.

In order to evaluate the impact of the network size, Fig 27 shows the found NMI for NG-SM in LFR networks with N = 1000 and N = 5000, the same parameters as in Fig 26, *p*_*l*_ = 0.9, *μ* = 0.1 and 0.3 and large communities—between 20 and 100 nodes.

Differently from the disjoint case, the size of the network does not have a significant influence on the accuracy obtained, and we see a high sensibility with relation to changes on the community structure of the network—*i.e.* smaller *μ*—given that the NMI values are much lower for networks with *μ* = 0.3.

**Fig 28:**
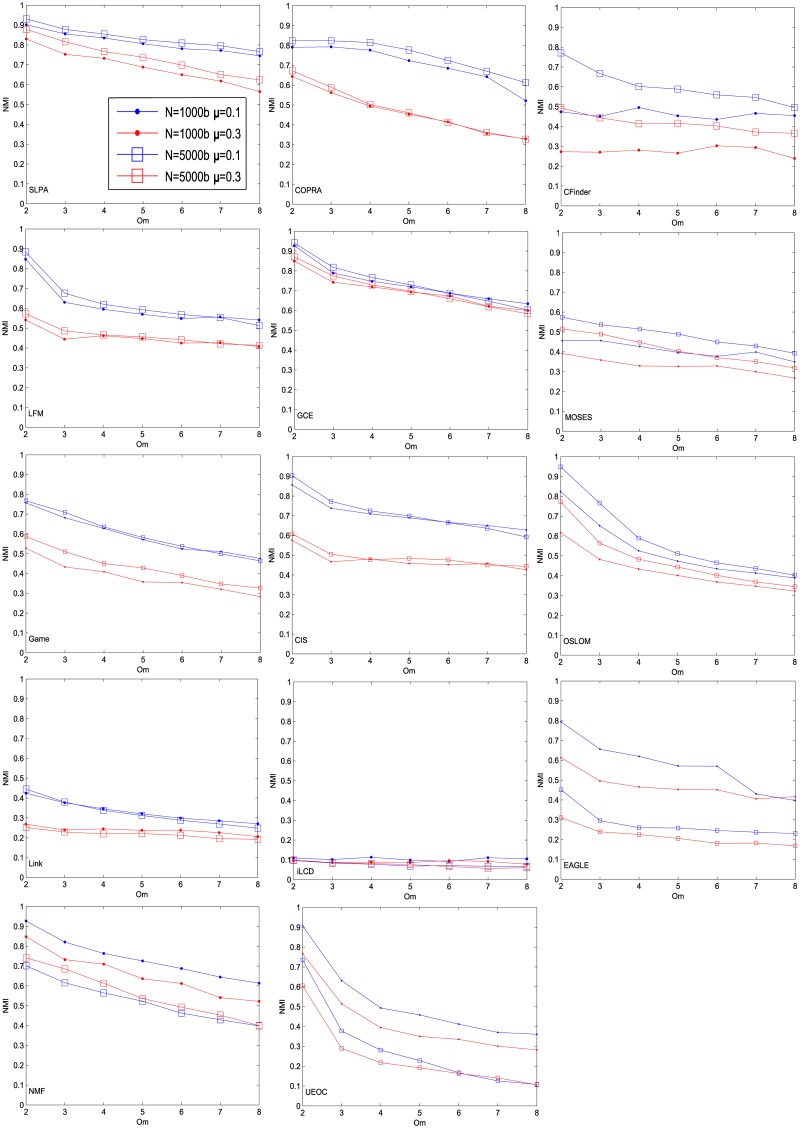
NMI for for different overlapping community methods, for comparison [23]. Reprinted with permission.

As in the last subsection, one can compare Fig 27 with NMI values for different existing algorithms with equivalent networks, as shown in Fig 28 [23]. Comparing NG-SM with other methods one can notice that our algorithm has good performance for networks with high community structure (*μ* = 0.1), being less sensible to the number of overlapping communities an agent can belong to than other algorithms. On the other hand, for networks with less community structure (*μ* = 0.3) the found NMI for NG-SM is better than most shown algorithms, like LFM, Cfinder and MOSES, but still presents worse accuracy when compared with other methods, like SLPA and GCE.

**Fig 29:**
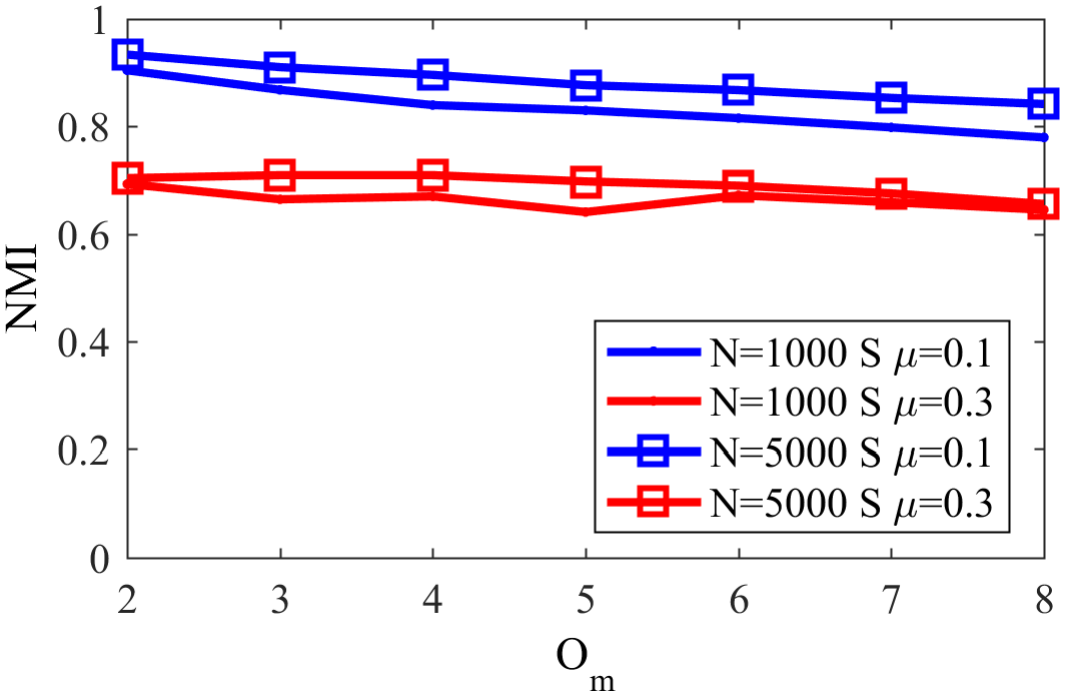
NMI overlap with small communities. NMI in LFR networks with overlapping communities of small size.

Finally, in order to analyze the influence of the size of communities, Fig 29 presents NMI values for networks with the same parameters but smaller communities (10 to 50 nodes). As in the disjoint case, we see that NG-SM has higher accuracy when detecting small communities, specially for the networks with *μ* = 0.3, with an approximate 15% NMI increase. In this sense, we can conclude that NG-SM in the overlapping case is better suited for networks with high community structure and communities of small size, noting that it also has a relative good performance in other tested networks.

Even though the values of NMI achieved with the proposed algorithm are not as good as for disjoint communities, meaning that more nodes in this case are misclassified, the results found here are comparable with many algorithms developed for detecting overlapping communities. As suggested earlier in this section, overlapping nodes have a different behavior than non-overlapping nodes due to the nature of the Naming Game, that permits only one successful word per interaction. For this matter, in the next section we briefly propose two adaptations of the proposed model that could improve the accuracy for modeling overlapping communities formation, and present the main conclusions of this work.

## Discussion and conclusion

In this work we have analyzed Community Detection methods based on local pairwise information exchange. For this matter social features were inserted in the Naming Game model, that led to the formation of word-sharing communities (or speech communities), in parallel with the sociological definition of [5]. The found communities are more similar to the topological communities as we increase the number of modeled social features.

The first inserted feature is trust, and we use the Naming Game based model presented in [8]—the Naming Game with Adaptive Weights (NG-AW)—as the model incorporating it. We show that, depending on the input parameter *ϵ*, it is possible to reach a meta-stable state where different groups of nodes sharing different words emerge, corresponding to different communities. Also, the resulting trust values separate the edges into internal and external ones. However, this only happens in simple networks with high community structure. For other networks consensus is reached in almost all executions.

The second modeled feature is uncertainty, related to the uncertainty the agent has in its values of trust defined so far, along a game execution. With this model—the Naming Game with Local Exploration Factor—we were able to reach non-convergence with higher probability and with less restrictions on the input parameter, compared to NG-AW. In addition, the values of uncertainty of nodes at the end of the game are related to their proportion of external edges, and the resulting edges’ weights divide them better into internal and external. Even though that happens for networks with community structure less obvious when compared to NG-AW, a little decrease on the community redundancy of the network leads to a large decrease on the accuracy of found communities, if any.

Finally it’s incorporated opinion preference, the third social feature, in the Naming Game with Secondary Memory (NG-SM). NG-SM improves the uncovering the communities in artificial networks generated with LFR benchmark—and networks that were intractable with the others NGs have low convergence probability with high NMI, thus leading to good accuracy in community detection. Moreover, the two classifications of nodes and edges with uncertainty and trust are more accurate and well divided.

The addition of a memory obviously increases the physical memory use in NG-SM, and some study on the impact of the second memory in this matter would be interesting. However, in cases with restriction of available memory, we argue that the elimination of low successful words from the secondary memories in middle and later stages of the game—thus reducing memory use burden—should not significantly interfere with the algorithm presented behavior.

We show that the network size influences the uncovering of communities, as larger networks will lead to a better detection. Also, the size of communities is another important parameter: NG-SM has better performance when applied to networks with smaller communities. Both behaviors are different from most CD algorithms, that tend to miss small communities and work better in smaller networks. This happens due to the local nature of the NG model, that uses peer-to-peer interactions to determine the communities, while other methods rely on global information. Our method also works better for denser networks, *i.e.* with higher average degree.

Due to the existence of various words and their successes counting in the secondary inventory, NG-SM was applied to overlapping communities. We show that overlapping nodes tend to have larger failure rate, due to receiving dominant words from the different communities they belong to. This results in lower *ϵ* for these nodes and worse classification of their edges, given that the trust values will be similar for all edges of an overlapping node. The responsible is the consensus-reaching idea of the Naming Game, on which an individual will agree in defining a single word per concept, and not more than one. In this matter, we suggest yet another alteration on the Naming Game we believe could bring some improvement on the accuracy of the model: enable success with multiple words, as if a node keeps changing from one word to others repetitively, it would have success with one of them without erasing the others it has success frequently too. This could be done by using the count of occurrences of the secondary memory in some trivial way, in medium and later stages of the game. In the social scope, as the rest of this work, this could be interpreted as a bilingual social feature, where people who speak more than one language have stored in their memories more than one word for each concept, and communicate successfully with all of them.

Another conclusion from the overlapping case is that two communities can be connected without any links between them, if a node belongs to both of them. In this case, an overlapping node can spread words from one community to others it belongs to, simply by being chosen as speaker. This would happen in an internal communication of an overlapping speaker, so it would not be enough to weight edges or words, given that a communication through a high trust edge and with a high weighted word also could lead to the spread of a word to a different community, and this would happen many times during the game, disturbing the dynamics. So, we describe another suggestion that could result in better accuracy in the model: The deactivation of speakers overlapping nodes—in this way, nodes will try to identify if they themselves are overlapping, and if they are, only communicate with a given probability. This would be harder to be interpreted as a social feature and can be seen as a heuristic procedure for a possible improvement on the method. Nevertheless, it is worth pointing out that NG-SM, as presented in this work, has accuracy comparable with other known algorithms for CD.

The NG-based models were not applied to hierarchical communities, where communities exist inside larger communities. We believe that in this case, the algorithm possibly would have different meta-stable states in time, presenting plateaus of *N*_*d*_ on the stages the communities could be found, like it happens on other dynamical algorithms. Some analysis on this issue could be interesting for further research.

Still on this subject of future work, some experiments could be made to verify if a community structure rises after (or during) the game played on a network without community structure, such as fully connected, random or lattice topologies. The last one could represent the potential connections of an individual due to its geographical position. We believe that these experiments could lead to some interesting insights in the matter of communities formation in a different, but still realistic way.
